# Vibration response of blue honeysuckle branches based on mathematical model

**DOI:** 10.1371/journal.pone.0354337

**Published:** 2026-07-23

**Authors:** Yuan Wei, Wang Ruiyin, Wang Yecheng, Feng Fang, Ma Decai

**Affiliations:** 1 College of Agricultural Equipment and Energy Engineering, Northeast Agricultural University, Harbin, China; 2 Sino-French Institute of Nuclear Engineering and Technology, Sun Yat-sen University, Zhuhai, China; China University of Mining and Technology, CHINA

## Abstract

For vibratory harvesting of blue honeysuckle, excitation must generate sufficient inertial force to detach the fruit, but the vibration energy is always absorbed by the trunk and root system, which decreases the inertial force. This study investigated the vibration response of fruit branches under excitation to achieve efficient harvesting. A simplified Y‑shaped branch model was established, and then dynamic equations of this branch system were derived. Based on branch angle characteristics, the dynamic equations were simplified. When the angle between a branch axis direction and the excitation direction is small, a specific excitation frequency induces parametric resonance in the branch system, leading to unstable vibration of the branch system. Under weak coupling conditions, the relationship among excitation amplitude, frequency, and geometric parameters of the branch that produce unstable vibration was derived, and the influence of variations in branch geometric parameters on the instability region was found. By establishing a finite element model of the branch system, the natural frequencies were calculated, and vibration amplitudes under different excitation frequencies were obtained through numerical simulation, which clarified the response characteristics of the branch during parametric resonance. To verify the theoretical and finite element analyses, branch vibration experiments were conducted. The maximum vibration amplitude of the branch under different excitation frequencies was measured, and the parametric resonance behavior was analyzed accordingly. The finite element calculations and experimental results agreed well with the theoretical model predictions, indicating that the theoretical model can provide a reliable basis for the design of vibratory harvesting machinery for blue honeysuckle.

## 1. Introduction

Blue honeysuckle (*Lonicera caerulea* L.) is a small berry, and manual harvesting is considerably difficult. Therefore, mechanized harvesting has become an inevitable trend in the development of harvesting methods. Among various mechanical harvesting techniques, vibratory harvesting has been confirmed as the most promising approach for engineering applications.

During vibratory harvesting, the excitation induces forced vibration in the fruit branches, imparting acceleration to the fruits. Consequently, inertial forces are generated, leading to fruit detachment. Numerous studies have theoretically established response models of the branch system and systematically analyzed the parameters affecting the dynamic response of fruits [1–3]. However, when vibratory harvesting is performed with external excitation, excessive vibration amplitude can easily cause bark tearing, xylem damage, and root loosening, thereby affecting the sprouting, flowering, and fruiting of the trees in the following year. Pu et al [4] pointed out that fruits are tender and fragile, making them prone to damage during picking, and trees can also be injured. Zhou et al [5] found that in jujube tree vibration, increasing fruit vibration amplitude raises the maximum stress in the stem during vibration, thereby increasing the risk of branch damage. Ortiz et al [6] recorded fruit motion during harvesting using high-speed video imaging and found that higher amplitude generated greater impact forces from vibration. The higher amplitude resulted in a higher damage grade. Liu et al [7] found that larger amplitude significantly increased the stress on the fruit stalks or branches of walnut trees. Gao et al [8] investigated the formation mechanism of mechanical damage in fruits and discussed the principles and recent advances in numerical simulation methods. Du et al [9] designed a three‑dimensional shaking mechanism with orthogonal eccentric blocks to reduce damage by adjusting the excitation pattern. Jia et al [10] systematically analyzed the dynamic response of fruits under different excitation modes and found that circular excitation is suitable for side branch harvesting, while combined excitation is more appropriate for whole tree harvesting. Collectively, these studies consistently provide evidence that amplitude is closely linked to damage.

The morphology of fruit branches is a key factor determining their vibration characteristics. Modeling fruit branches is crucial for understanding the vibratory harvesting process. Jiao et al [11] employed the concentrated mass method to construct a differential vibration model of a branch and pointed out that selecting the maximum peak frequency in the trunk vibration spectrum as the excitation frequency achieves the best fruit detachment effect. Zhuo et al [12] analyzed the vibratory harvesting mechanism of jujube trees and established a transverse bending vibration mechanics model of jujube branches. Lang et al [13] constructed a common model composed of two Maxwell elements, a damping element and a spring, all coupled in parallel. Chen et al [14] built three‑dimensional models of branches, ripe fruits, unripe fruits, flowers, and leaves, and assembled them into a branch‑fruit‑flower‑leaf system model based on the growth characteristics of Lycium barbarum L. Wang et al [15] established dynamic equations for fruit branch picking, and mathematically solved the forced vibration output solution and picking inertial force for camellia fruit. Yuan et al [16] developed a dynamic model for the blue honeysuckle fruit-stem system at a certain angle to the excitation direction. Mei et al [17] studied the vibratory picking principle of Chinese wolfberry branches and established a mechanical model for vibratory picking based on a simplified cantilever beam model. Liu et al [18] developed a dynamic model of a camellia tree branch‑fruit system based on an epitrochoid trajectory, identifying vibration frequency and trajectory amplitude as key detachment factors.

Sola-Guirado et al [19] investigated the influence of leaves on the dynamic response of olive branches and established a corresponding computational model. Song et al [20] studied the abscission mechanics model of a peach fruit–branch system by combining a mixed-mode cohesive zone model (CZM) with the finite element method (FEM), focusing on the failure mechanism of the fruit detachment zone. Gao et al [21] developed and experimentally validated a handheld vibratory harvesting device intended for Camellia oleifera fruit harvesting. Chen et al [22] simulated the bending morphology of Lycium barbarum fruit-bearing branches using FEM, concentrating on the deformation fitting of the branch under load. Liu et al [23] investigated a multi-degree-of-freedom (MDOF) dynamic simulation model for the vibratory harvesting of Prunus cerasifera, combining computational and experimental analyses. He et al [24] studied the effect of fruit position on apple detachment under mechanical vibration, focusing on detachment thresholds or detachment efficiency. Wang et al [25] investigated the detachment patterns and impact characteristics of litchi under vibratory harvesting, addressing how fruit detaches and its force conditions.

Sun et al [26] examined the vibration response characteristics and separation-deformation behavior of fruit-branch systems, focusing on the detachment mechanism in harvesting. Li et al [27] predicted the inertial forces on Camellia oleifera fruit during vibratory harvesting using machine learning and SHAP explanations, representing an intelligent parameter prediction study. Jiao et al [28] designed and tested a dual-symmetric eccentric exciter to support the development of vibratory harvesting equipment for fruit trees. Ru et al [29] constructed and experimentally analyzed a response model for a walnut vibratory harvesting system. Castro-Garcia et al [30] evaluated the suitability of Spanish ‘Manzanilla’ table olive orchards for trunk shaking harvesting. Pu et al [31] studied the selection and experimental evaluation of canopy shaker clamps, with the objective of reducing tree damage during citrus harvesting. Sola-Guirado et al [32] investigated an olive harvester that simultaneously shakes the trunk and branches. Zhang et al [33] studied the influence of pruning strategies on the effectiveness of vibratory apple harvesting and experimentally analyzed the vibration response of fruit-bearing branches of Lycium barbarum. Castro-Garcia et al [34] analyzed the detachment process of late-season ‘Valencia’ oranges during canopy shaking harvesting. Wei et al [35] combined rigid-flexible coupling simulation with experiments to analyze the vibration characteristics of pistachio trees for optimizing mechanical harvesting efficiency. Ortiz et al [36] determined the appropriate frequency, amplitude, and duration required for fresh citrus mechanical harvesting. Dang et al [37] optimized the vibratory harvesting process of olive trees by integrating response surface methodology with rigid–flexible coupling simulation.

To minimize mechanical damage to branches and fruits, it is necessary to systematically analyze the energy transfer pattern among different branches and accurately determine the vibration energy threshold required for fruit detachment. Castro‑Garcia et al [38] applied forced vibration and measured branch responses using triaxial accelerometers. They found that acceleration attenuates significantly with distance, and that leaves substantially increase damping. Zheng et al [39] reported that during vibration of winter jujube, at non‑resonant frequencies, the acceleration response of most branches gradually increases from base to tip. Lin et al [40] pointed out that in a study on ginkgo tree vibration, at low frequencies, the acceleration response is very weak; at higher frequencies, the vibration response becomes stronger but exhibits different characteristics among different branches, with the acceleration amplitude on the trunk always being the smallest.

Previously, researchers developed a mathematical model to analyze the vibration response characteristics of blue honeysuckle branches [16]. However, it only considered the response of a single fruit branch under a uniformly distributed load, without accounting for the complex morphology of the branch system or the response of such a system under vibratory excitation. Many dynamic models of fruit branches treat each branch as a rigid body connected by spring and damper elements. Neglecting geometric features such as branch length and angle leads to insufficient accuracy of the model. For the basic bifurcation unit of a fruit branch, Tang et al [41] modeled it as a cantilevered trunk and obtained the modal frequencies without considering the plant mass; however, the bifurcation angle was held constant. To precisely investigate the vibration response of fruit branches under external excitation, this study adopts a more refined modeling approach. A Y-shaped fruit branch is taken as a three-branch system for vibration analysis. Since the natural frequencies corresponding to the rigid body rotational modes of slender structures are significantly lower than the natural frequencies of bending deformation, and low‑order natural frequencies dominate vibratory harvesting, only the rotational modes of the branches are considered in this model. The three branches of the Y-shaped branch are treated as cylindrical rigid bodies, and are interconnected with torsional springs and rotational dampers. The three branches in the branch system are defined as the main stem, the upper stem, and the side branch. Under a periodic excitation force applied to the root of the main stem, the rotational motions of the three branches about their connecting joints are considered, and the dynamic equations of the branch system are established. Finally, the equations are simplified based on branch angle characteristics.

When the angle between the side branch axis and the excitation direction is small, the dynamic equations of the system can be reduced to parametric resonance equations. Under weakly coupled conditions, these parametric resonance equations can be transformed into the standard form of the Mathieu equation. When the excitation frequency is close to twice the system’s natural frequency, an instability region appears in the branch system. Based on the analysis of the mass matrix and the parametric excitation matrix, combined with the stability theory of the Mathieu equation, the instability regions of the branch system under different excitation frequencies and amplitudes can be determined. Considering the complexity of branch morphology, the vibration stability of the branch system with different geometric features is studied in a unified manner using the mass ratios and length ratios. Using Mathematica, the variation boundaries of the instability region are plotted as functions of the mass ratios and length ratios, as well as to different lengths of the side branch and the upper stem, are plotted. A finite element model of the branch system is established to obtain the system’s natural frequencies, and the branch system is excited at a frequency equal to twice the lowest natural frequency to study its parametric resonance behavior. Finally, vibration experiments are conducted on blue honeysuckle branches. The amplitude responses of the side branches under different excitation frequencies are used to verify the proposed theoretical model. The experimental results show that a smaller excitation amplitude can induce large vibration amplitude of the branch system.

This study establishes a parametric resonance model for a Y-shaped branch structure modeled as a rigid-body-torsional-spring system, which better captures the dynamic coupling between branches. It reveals the essential difference between parametric resonance and ordinary resonance in vibratory harvesting, and demonstrates that parametric resonance can achieve large-amplitude branch response, which is a unique engineering advantage for energy-efficient and tree-friendly harvesting. Based on a systematic parameter sensitivity analysis, we provide direct engineering recommendations for selecting the optimal excitation frequency and controlling side branch geometry, bridging the gap between theoretical foundation and mechanical harvesting equipment.

## 2. Materials and methods

### 2.1 Materials

The experiments were conducted in June 2025 at the Horticultural Experiment Station of Northeast Agricultural University in Heilongjiang Province, China, using healthy and vigorous blue honeysuckle plants (*Lonicera caerulea* L.; 5–8 years old). Ten Y-shaped branch samples of similar size were randomly selected and sequentially numbered. The experiments were conducted on June 12, 2025, and lasted five days.

The diameter was measured using a digital vernier caliper (Model: CJW888, resolution: 0.02 mm). Tensile tests were conducted on an ST-series universal testing machine, specifically the 1ST single-column model (Model: 1ST-50ST, manufactured by Tinius Olsen, USA), which has a speed accuracy of ±0.05% and a load range of 0-1kN. A force sensor was integrated into the testing system. The obtained parameters are presented in Table 1 [16]. The vibration actuator employed an eccentric crank–slider mechanism developed by our research group. The rotational speed of the eccentric mechanism was controlled by a frequency converter (Model: DELTA VFD007M43B, frequency range: 0–60 Hz) to regulate the motor speed. The vibration amplitude was adjusted by changing the position of the connecting rod on the eccentric wheel. High-speed imaging was performed using a Phantom VEO 4K 990L camera (manufactured by VRL, Vision Research Inc., USA) with a resolution of 1024 × 1024 pixels and a frame interval of 1667µs per frame. Vibration amplitude analysis was carried out via post‑processing using the PCC3.12 (Phantom Camera Control) high‑speed imaging software.

**Table 1:** Basic parameters of blue honeysuckle.

Age (year)	5-8
Density of the fruit branch ρ ($\text{kg}\cdot {\text{m}}^{-\text{3}}$)	887
Elastic modulus of the fruit branch E (Mpa)	5023
Poisson’s ratio	0.3

Values presented are mean.

### 2.2 Method

**Fig 1:**
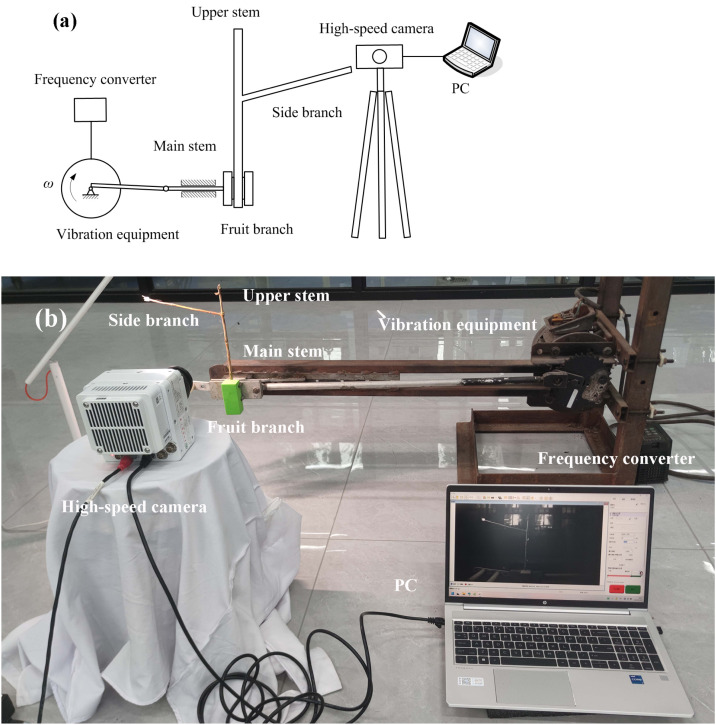
Schematic diagram of the high-speed camera-based vibration test. (a) Schematic diagram (b) Actual experimental scene diagram. Note: This is an original figure created by the authors.

During vibration testing, a clamp was used to keep the end of the main stem upright, and then vibration excitation was applied. The maximum vibration amplitude of the side branch in the vertical direction was measured by a high-speed camera. A schematic diagram of the test setup is shown in Fig 1(a) and the actual experimental scene is shown in Fig 1(b). The rotational speed of the drive motor was adjusted by a frequency converter to control the excitation frequency of the eccentric linkage mechanism. The vibration amplitude was controlled by altering the relative installation position between the connecting rod and the eccentric wheel. The rotational speed was tested from 300 to $\text{3000r}\cdot {\text{min}}^{-1}$. The vibration amplitude was maintained at 20 mm. The excitation time was set at 5s. A total of 10 test samples were tested, and for each vibration frequency, the maximum amplitude of the side branch tip in the vertical direction was recorded.

#### 2.2.1 Mathematical model of blue honeysuckle branches.

**Fig 2:**
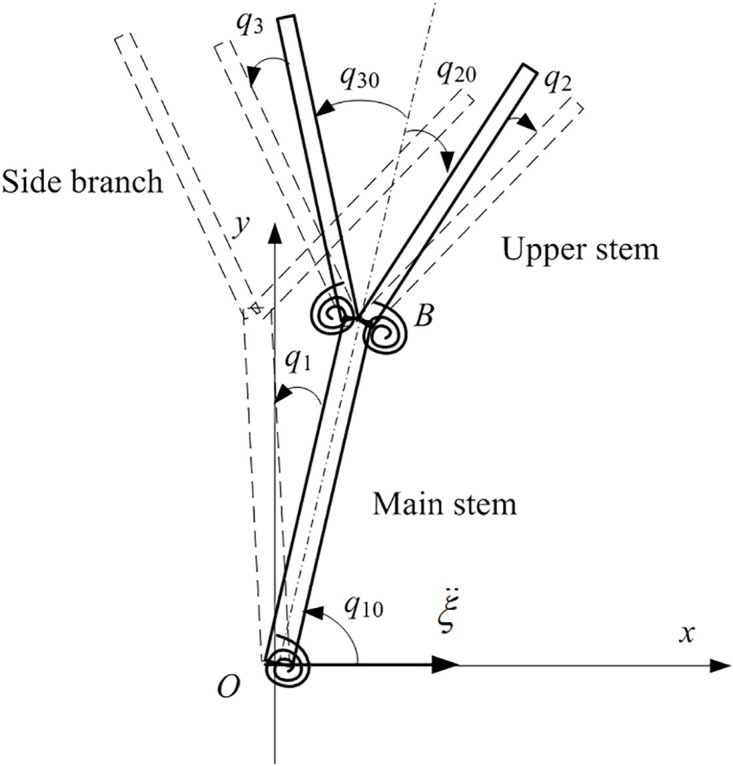
Schematic diagram of vibration of Y-shaped fruit branch.

Blue honeysuckle exhibits a complex structure, which greatly complicates modeling of the overall structure. During vibratory harvesting, the excitation point is located on the main stem. If the influence of the stem below the excitation point is neglected, then the mathematical modeling can be performed only on the upper part of the blue honeysuckle branch, enabling further investigation of the vibration response of this upper part. Considering a Y-shaped structure of the blue honeysuckle branch, a schematic diagram of the simplified structure is shown in Fig 2. The excitation applied to the fruit branch can be considered imposed on the base of the main stem.

Consider the Y-shaped structure as a fruit branch system, consisting of a main stem, an upper stem, and a side branch, all three of which are regarded as rigid cylindrical bodies and lie within a single plane. Each pair of adjacent branches is connected by a torsional spring and a damper, allowing rotational motion. The entire branch system vibrates under the excitation $\ddot{\xi }$ at joint O. For a slender cantilever structure, the natural frequency corresponding to the rotational mode about its ends is significantly lower than the fundamental frequency of its bending vibration. Low natural frequencies typically correspond to the modes that are easily excited in the system, so for vibration harvesting, low natural frequencies are very important. Therefore, the system shown in Fig 2 can represent the rotational behavior of the three branches and suitably describe the low-order modes of the branch system vibration.

The basic assumptions of this study are as follows: the Y-shaped branch lies in the same plane as the excitation force; each fruit-bearing branch is modeled as a homogeneous circular cylinder; and small deformation conditions are satisfied. In vibration harvesting, the excitation force is applied directionally. If the plane of a Y-shaped fruit branch happens to lie almost within the plane of this force application, the majority of the excitation energy is directly transmitted into that plane. A Y-shaped fruit branch can be regarded as a planar frame structure. Compared with in-plane bending, an out-of-plane deformation that displaces the branch to a given position is resisted jointly by bending stiffness and torsional stiffness. The system thus incorporates both a bending spring and a torsional spring, and consequently possesses a higher overall stiffness coefficient than a system with only a torsional spring. This means the out-of-plane bending stiffness of the branch is substantially larger than its in-plane bending stiffness.

The branch system has low in-plane stiffness. Out-of-plane vibration requires branch twisting or lateral swaying, which involve high stiffness. Consequently, the first-order natural frequency of out-of-plane vibration is higher than that of in-plane vibration. When the excitation frequency is low, the out-of-plane response is therefore very small compared with the in-plane response. Hence, at low excitation frequencies, the maximum vibration amplitude of the fruit branch occurs in that plane, where the stiffness is lower and the external input energy is primarily transmitted into the in-plane motion. Fruit detachment depends on the inertial force experienced by the fruit. Because the excitation force is directionally fixed, the dominant component of the inertial force acting on the fruit is generated within the plane of the Y-shaped fruit branch.

In addition, the fruit-bearing branches are simplified as homogeneous circular cylinders. Measurements show that most branches of blue honeysuckle exhibit small diameter variations, nearly circular cross-sections, and a uniform density distribution. Adopting a homogeneous cylinder model captures the main geometric and inertial characteristics of the branches while avoiding unnecessary local details. Furthermore, large deformations of the branches have little effect on their low-order natural frequencies; therefore, only the small deformation case is considered here; it is assumed that the displacements of the branches are smaller than their characteristic length. Under this condition, there is no need to distinguish between the undeformed and deformed configurations when establishing the equilibrium equations.

Hence, this study considers only the exciting force and the branch system to be confined to a single plane. The dynamic equations for the simplified mechanical model in Fig 2 are established as follows. Specifically, three angular momentum equations are written for joints O and B of the branch system. Following the method in [42] the angular momentum equation for the three branches acting together on joint O can be written as:


$$\begin{array}{c} \begin{array}{c} \left[\frac{\text{1}}{\text{3}}m_{\text{1}}l_{\text{1}}^{\text{2}}+\frac{\text{1}}{\text{3}}m_{\text{2}}l_{\text{2}}^{\text{2}}+m_{\text{2}}l_{\text{1}}^{\text{2}}-m_{\text{2}}l_{\text{1}}l_{\text{2}}cos\left(q_{\text{2}}\text{+}q_{2\text{0}}\right)+\frac{\text{1}}{\text{3}}m_{\text{3}}l_{\text{3}}^{\text{2}}+m_{\text{3}}l_{\text{1}}^{\text{2}}-m_{\text{3}}l_{\text{1}}l_{\text{3}}cos\left(q_{\text{3}}\text{+}q_{\text{30}}\right)\right]{\ddot{q}}_{\text{1}}\\ +\left[\frac{\text{1}}{\text{3}}m_{\text{2}}l_{\text{2}}^{\text{2}}-\frac{\text{1}}{\text{2}}m_{\text{2}}l_{\text{1}}l_{\text{2}}cos\left(q_{\text{2}}\text{+}q_{2\text{0}}\right)\right]{\ddot{q}}_{\text{2}}+\left[\frac{\text{1}}{\text{3}}m_{\text{3}}l_{\text{3}}^{\text{2}}-\frac{\text{1}}{\text{2}}m_{\text{3}}l_{\text{1}}l_{\text{3}}cos\left(q_{\text{3}}\text{+}q_{\text{30}}\right)\right]{\ddot{q}}_{\text{3}}\\ -\frac{\text{1}}{\text{2}}m_{\text{2}}l_{\text{1}}l_{\text{2}}sin\left(q_{\text{2}}\text{+}q_{2\text{0}}\right)\left(2{\dot{q}}_{\text{1}}{\dot{q}}_{\text{2}}+{\dot{q}}_{\text{2}}^{\text{2}}\right)-\frac{\text{1}}{\text{2}}m_{\text{3}}l_{\text{1}}l_{\text{3}}sin\left(q_{\text{3}}\text{+}q_{\text{30}}\right)\left(2{\dot{q}}_{\text{1}}{\dot{q}}_{\text{3}}+{\dot{q}}_{\text{3}}^{\text{2}}\right)\\ -\left(\frac{\text{1}}{\text{2}}m_{\text{1}}l_{\text{1}}+l_{\text{1}}(m_{\text{2}}+m_{\text{3}})\right)\left(\ddot{\xi }sin\left(q_{\text{1}}\text{+}q_{\text{10}}\right)-gcos\left(q_{\text{1}}\text{+}q_{\text{10}}\right)\right)\\ +\frac{\text{1}}{\text{2}}m_{\text{2}}l_{\text{2}}\left(\ddot{\xi }sin\left(q_{\text{1}}\text{+}q_{\text{10}}+q_{\text{2}}\text{+}q_{\text{20}}\right)-gcos\left(q_{\text{1}}\text{+}q_{\text{10}}+q_{\text{2}}\text{+}q_{\text{20}}\right)\right)\\ +\frac{\text{1}}{\text{2}}m_{\text{3}}l_{\text{3}}\left(\ddot{\xi }sin\left(q_{\text{1}}\text{+}q_{\text{10}}+q_{\text{3}}\text{+}q_{\text{30}}\right)-gcos\left(q_{\text{1}}\text{+}q_{\text{10}}+q_{\text{3}}\text{+}q_{\text{30}}\right)\right)+k_{\text{1}}q_{\text{1}}+c_{\text{1}}{\dot{q}}_{\text{1}}=\text{0} \end{array} \end{array}$$ (1)

The angular momentum equation of the upper stem with respect to the joint B is:


$$\begin{array}{c} \begin{array}{c} \left[\frac{\text{1}}{\text{3}}m_{\text{2}}l_{\text{2}}^{\text{2}}-\frac{\text{1}}{\text{2}}m_{\text{2}}l_{\text{1}}l_{\text{2}}cos\left(q_{\text{2}}\text{+}q_{2\text{0}}\right)\right]{\ddot{q}}_{\text{1}}+\frac{\text{1}}{\text{3}}m_{\text{2}}l_{\text{2}}^{\text{2}}{\ddot{q}}_{\text{2}}+\frac{\text{1}}{\text{2}}m_{\text{2}}l_{\text{1}}l_{\text{2}}sin\left(q_{\text{2}}\text{+}q_{2\text{0}}\right){\dot{q}}_{\text{1}}^{\text{2}}\\ -\frac{\text{1}}{\text{2}}m_{\text{2}}l_{\text{2}}\left(\ddot{\xi }sin\left(q_{\text{1}}\text{+}q_{\text{10}}+q_{\text{2}}\text{+}q_{2\text{0}}\right)-gcos\left(q_{\text{1}}\text{+}q_{\text{10}}+q_{\text{2}}\text{+}q_{2\text{0}}\right)\right)+k_{\text{2}}q_{\text{2}}+c_{\text{2}}{\dot{q}}_{\text{2}}=\text{0} \end{array} \end{array}$$ (2)

The angular momentum equation of the side branch about the joint B is:


$$\begin{array}{c} \begin{array}{c} \left[\frac{\text{1}}{\text{3}}m_{\text{3}}l_{\text{3}}^{\text{2}}-\frac{\text{1}}{\text{2}}m_{\text{3}}l_{\text{1}}l_{\text{3}}cos\left(q_{\text{3}}\text{+}q_{\text{30}}\right)\right]{\ddot{q}}_{\text{1}}+\frac{\text{1}}{\text{3}}m_{\text{3}}l_{\text{3}}^{\text{2}}{\ddot{q}}_{\text{3}}+\frac{\text{1}}{\text{2}}m_{\text{3}}l_{\text{1}}l_{\text{3}}sin\left(q_{\text{3}}\text{+}q_{\text{30}}\right){\dot{q}}_{\text{1}}^{\text{2}}\\ -\frac{\text{1}}{\text{2}}m_{\text{3}}l_{\text{3}}\left(\ddot{\xi }sin\left(q_{\text{1}}\text{+}q_{\text{10}}+q_{\text{3}}\text{+}q_{\text{30}}\right)-gcos\left(q_{\text{1}}\text{+}q_{\text{10}}+q_{\text{3}}\text{+}q_{\text{30}}\right)\right)+k_{\text{3}}q_{\text{3}}+c_{\text{3}}{\dot{q}}_{\text{3}}=\text{0} \end{array} \end{array}$$ (3)

where $\ddot{\xi }$ is the external excitation acceleration, $q_{1\text{0}}$ is the initial angle between the main stem axis and the horizontal direction, $q_{\text{20}}$ is the initial angle between the upper stem axis and the main stem axis, $q_{\text{30}}$ is the initial angle between the side branch axis and the main stem axis, $q_{\text{1}},q_{\text{2}},q_{\text{3}}$ represent the angular displacements of the three fruit branches, $c_{\text{1}},c_{\text{2}},c_{\text{3}}$ represent the damping coefficients associated with the three fruit branches, $m_{\text{1}},m_{\text{2}},m_{\text{3}}$ represent the masses of the three fruit branches, $l_{\text{1}},l_{\text{2}},l_{\text{3}}$ represent the lengths of the three fruit branches, $k_{\text{1}},k_{\text{2}},k_{\text{3}}$ represent the spring constants of the torsion springs connecting the three fruit branches, and g is the gravitational acceleration.

#### 2.2.2 Simplification of the dynamic equations.

The dynamic equations can be simplified using the actual angles of fruit branches. During vibratory harvesting, the main stem is typically perpendicular to the ground, so it is assumed that $q_{1\text{0}}=\pi \text{/}2$. Although the clamping device can constrain the initial angle of the main stem, it cannot restrict its dynamic variation. Therefore, it is reasonable to assume that $q_{\text{1}}$, as well as ${\dot{q}}_{\text{1}}$ and ${\ddot{q}}_{\text{1}}$, all undergo changes. Assuming the vibration angles of the fruit branches are small, $q_{\text{1}},q_{\text{2}},q_{\text{3}}\ll 1$. At the initial moment, the main stem and upper stem are assumed to be aligned, yielding $q_{20}=0$. Furthermore, assuming the side branch is initially perpendicular to the main stem, we have $q_{30}=\pi \text{/2}$. Based on the above initial branching angles and the small angle vibration assumption, further linearization is required to simplify the three dynamic equations. By neglecting higher-order terms such as $q_{i}q_{j}$, ${\dot{q}}_{i}{\dot{q}}_{j}$, $q_{i}^{\text{2}}$, and expanding the sine and cosine functions as a first-order Taylor series around the equilibrium point, Eq. (1) is simplified to:


$$\begin{array}{c} \begin{array}{c} \left(\frac{\text{1}}{\text{3}}m_{\text{1}}l_{\text{1}}^{\text{2}}+\frac{\text{1}}{\text{3}}m_{\text{2}}l_{\text{2}}^{\text{2}}+m_{\text{2}}l_{\text{1}}^{\text{2}}-m_{\text{2}}l_{\text{1}}l_{\text{2}}+\frac{\text{1}}{\text{3}}m_{\text{3}}l_{\text{3}}^{\text{2}}+m_{\text{3}}l_{\text{1}}^{\text{2}}\right){\ddot{q}}_{\text{1}}+\left(\frac{\text{1}}{\text{3}}m_{\text{2}}l_{\text{2}}^{\text{2}}-\frac{\text{1}}{\text{2}}m_{\text{2}}l_{\text{1}}l_{\text{2}}\right){\ddot{q}}_{\text{2}}\\ +\frac{\text{1}}{\text{3}}m_{\text{3}}l_{\text{3}}^{\text{2}}{\ddot{q}}_{\text{3}}+c_{\text{1}}{\dot{q}}_{\text{1}}+\left[k_{\text{1}}-\left(\frac{\text{1}}{\text{2}}m_{\text{1}}l_{\text{1}}+m_{\text{2}}l_{\text{1}}+m_{\text{3}}l_{\text{1}}+\frac{\text{1}}{\text{2}}m_{\text{2}}l_{\text{2}}\right)g+\frac{\text{1}}{\text{2}}m_{\text{3}}l_{\text{3}}\ddot{\xi }\right]q_{\text{1}}\\ -\frac{\text{1}}{\text{2}}m_{\text{2}}l_{\text{2}}gq_{\text{2}}+\frac{\text{1}}{\text{2}}m_{\text{3}}l_{\text{3}}\ddot{\xi }q_{\text{3}}=\left[\frac{\text{1}}{\text{2}}m_{\text{1}}l_{\text{1}}+l_{\text{1}}(m_{\text{2}}+m_{\text{3}})+\frac{\text{1}}{\text{2}}m_{\text{2}}l_{\text{2}}\right]\ddot{\xi }+\frac{\text{1}}{\text{2}}m_{\text{3}}l_{\text{3}}g \end{array} \end{array}$$ (4)

Eq. (2) is simplified to:


$$\begin{array}{c} \left(\frac{\text{1}}{\text{3}}m_{\text{2}}l_{\text{2}}^{\text{2}}-\frac{\text{1}}{\text{2}}m_{\text{2}}l_{\text{1}}l_{\text{2}}\right){\ddot{q}}_{\text{1}}+\frac{\text{1}}{\text{3}}m_{\text{2}}l_{\text{2}}^{\text{2}}{\ddot{q}}_{\text{2}}+c_{\text{2}}{\dot{q}}_{\text{2}}-\frac{\text{1}}{\text{2}}m_{\text{2}}l_{\text{2}}gq_{\text{1}}+\left(k_{\text{2}}-\frac{\text{1}}{\text{2}}m_{\text{2}}l_{\text{2}}g\right)q_{\text{2}}=\frac{\text{1}}{\text{2}}m_{\text{2}}l_{\text{2}}\ddot{\xi } \end{array}$$ (5)

Eq. (3) is simplified to:


$$\begin{array}{c} \frac{\text{1}}{\text{3}}m_{\text{3}}l_{\text{3}}^{\text{2}}{\ddot{q}}_{\text{1}}+\frac{\text{1}}{\text{3}}m_{\text{3}}l_{\text{3}}^{\text{2}}{\ddot{q}}_{\text{3}}+c_{\text{3}}{\dot{q}}_{\text{3}}+\frac{\text{1}}{\text{2}}m_{\text{3}}l_{\text{3}}\ddot{\xi }q_{\text{1}}+\left(k_{\text{3}}+\frac{\text{1}}{\text{2}}m_{\text{3}}l_{\text{3}}\ddot{\xi }\right)q_{\text{3}}=\frac{\text{1}}{\text{2}}m_{\text{3}}l_{\text{3}}g \end{array}$$ (6)

For the three equations Eqs. (4)–(6), and of the linearized system, a unified expression can be written in standard matrix form as:


$$\begin{array}{c} M_{b}\ddot{q}+C_{b}\dot{q}+\left(K_{b0}+\ddot{\xi }D_{b}\right)q=F_{b}\left(t\right) \end{array}$$ (7)

where $q=[q_{\text{1}},q_{\text{2}},q_{\text{3}}{]}^{\text{T}}$ is the vector of generalized coordinates, $M_{b}$ is the mass matrix, $C_{b}$ is the damping matrix, $K_{b0}$ is the constant stiffness matrix, and $\ddot{\xi }D_{b}$ is the parametric excitation matrix. When the excitation displacement is set to Acos(Ωt), $\ddot{\xi }$ can be expressed as $\ddot{\xi }=-A\Omega^{\text{2}}cos\left(\Omega t\right)$, where A is the amplitude of the excitation and Ω is the excitation frequency. $M_{b}=\left[\begin{array}{ccc} @ccc@M_{11} & M_{12} & M_{13}\\ M_{12} & M_{22} & 0\\ M_{13} & \;\;0 & M_{33} \end{array}\right]$, $C_{b}=\left[\begin{array}{ccc} @ccc@c_{\text{1}} & 0 & 0\\ 0 & c_{\text{2}} & 0\\ 0 & 0 & c_{\text{3}} \end{array}\right]$, $K_{b0}=\left[\begin{array}{ccc} @ccc@k_{\text{1}}-F_{\text{1}}g & -\frac{\text{1}}{\text{2}}m_{\text{2}}l_{\text{2}}g & \text{0}\\ -\frac{\text{1}}{\text{2}}m_{\text{2}}l_{\text{2}}g & k_{\text{2}}-\frac{\text{1}}{\text{2}}m_{\text{2}}l_{\text{2}}g & 0\\ \text{0} & 0 & k_{\text{3}} \end{array}\right]$, $\ddot{\xi }D_{b}=\frac{\text{1}}{\text{2}}\ddot{\xi }m_{\text{3}}l_{\text{3}}\left[\begin{array}{ccc} @ccc@1 & 0 & 1\\ 0 & 0 & 0\\ 1 & 0 & \text{1} \end{array}\right]$, $F_{b}\left(t\right)=\left[\begin{array}{c} @c@F_{\text{1}}\ddot{\xi }+\frac{\text{1}}{\text{2}}m_{\text{3}}l_{\text{3}}g\\ \frac{\text{1}}{\text{2}}m_{\text{2}}l_{\text{2}}\ddot{\xi }\\ \frac{\text{1}}{\text{2}}m_{\text{3}}l_{\text{3}}g \end{array}\right]$, $F_{\text{1}}=\frac{\text{1}}{\text{2}}m_{\text{1}}l_{\text{1}}+l_{\text{1}}(m_{\text{2}}+m_{\text{3}})+\frac{\text{1}}{\text{2}}m_{\text{2}}l_{\text{2}}$, $M_{11}=\frac{\text{1}}{\text{3}}m_{\text{1}}l_{\text{1}}^{\text{2}}+m_{\text{2}}\left(\frac{\text{1}}{\text{3}}l_{\text{2}}^{\text{2}}+l_{\text{1}}^{\text{2}}-l_{\text{1}}l_{\text{2}}\right)+m_{\text{3}}\left(\frac{\text{1}}{\text{3}}l_{\text{3}}^{\text{2}}+l_{\text{1}}^{\text{2}}\right)$, $M_{12}=m_{\text{2}}\left(\frac{\text{1}}{\text{3}}l_{\text{2}}^{\text{2}}-\frac{\text{1}}{\text{2}}l_{\text{1}}l_{\text{2}}\right)$, $M_{13}=M_{33}=\frac{\text{1}}{\text{3}}m_{\text{3}}l_{\text{3}}^{\text{2}}$, $M_{22}=\frac{\text{1}}{\text{3}}m_{\text{2}}l_{\text{2}}^{\text{2}}$.

In Eq. (4), The term $M_{12}\text{=}\frac{\text{1}}{\text{3}}m_{\text{2}}l_{\text{2}}^{\text{2}}-\frac{\text{1}}{\text{2}}m_{\text{2}}l_{\text{1}}l_{\text{2}}$ represents the inertial moment acting on the main stem caused by the angular acceleration of the upper stem ${\ddot{q}}_{\text{2}}$. The magnitude of this moment depends on the mass distribution and geometric dimensions of the upper stem. The first term, $\frac{\text{1}}{\text{3}}m_{\text{2}}l_{\text{2}}^{\text{2}}$, is the contribution of the moment of inertia of the upper stem about point B to the couple moment on the main stem. And the second term, $-\frac{\text{1}}{\text{2}}m_{\text{2}}l_{\text{1}}l_{\text{2}}$, is the moment exerted on the main stem by the inertial force, resulting from the translational acceleration of the center of mass of the upper stem.

In Eq. (6), the physical origin of the off-diagonal elements $\frac{\text{1}}{\text{2}}m_{\text{3}}l_{\text{3}}\ddot{\xi }q_{\text{1}}$ lies in the fact that the horizontal base vibration generates an inertial force $m_{\text{3}}\ddot{\xi }$ at the center of mass of the side branch. The horizontal vibration acceleration of the base $\ddot{\xi }$, together with the rotation angle $q_{\text{1}}$ of the main stem, modifies the moment arm length of the inertial force on the side branch. Specifically, the moment arm length becomes $\frac{\text{1}}{\text{2}}l_{\text{3}}q_{\text{1}}$, thereby producing a moment in the side branch that is proportional to $q_{\text{1}}$ and varies periodically with time.

#### 2.2.3 Dynamic equations based on proportional relationships.

The morphology of fruit branches varies significantly, with considerable differences in mass and length among branches. To analyze the vibrational responses of differently shaped branches, the dynamic equations are rewritten using dimensionless proportional relationships between branches. Let the reference mass be $m=m_{\text{1}}$ and the reference length be $l=l_{\text{1}}$. Accordingly, the masses and lengths of the upper stem and the side branch can be expressed as $m_{\text{2}}=a_{\text{2}}m$, $m_{\text{3}}=a_{\text{3}}m$, $l_{\text{2}}=b_{\text{2}}l$, and $l_{\text{3}}=b_{\text{3}}l$ respectively, where $a_{\text{2}}$ and $a_{\text{3}}$ are the mass ratios of the upper stem and the side branch to the main stem, $b_{\text{2}}$, $b_{\text{3}}$ are the corresponding length ratios.

The torsional spring stiffness of each fruit branch, $k_{\text{1}},k_{\text{2}},k_{\text{3}}$,can be approximately determined based on the elastic modulus of the fruit branch material. To find the equivalent torsional stiffness, the bending moment required at the root to produce a unit rotation at the tip must be determined. Assuming that the fruit branch to be a rigid body, and the torsional spring stiffness coefficient K represents the elasticity of the branch. Under an external force, the tip displacement arises solely from the rotation of the torsional spring. If a concentrated force F acts at the tip, the moment at the root is M=Fl, the rotation angle of the spring is θ=Fl/K, and the lateral displacement at the tip is $\theta l=Fl^{\text{2}}/K$.

When the fruit branch is treated as an elastic body, let E be the elastic modulus of the fruit branch, l be the length of the fruit branch, and $I_{b}$ be the moment of inertia of its cross section, $EI_{b}$ be the bending stiffness of the fruit branch. If the fruit branch is modeled as a cantilever beam, the deflection at its tip under a force F is $y=\frac{Fl^{\text{3}}}{3EI_{b}}$. The lateral tip displacement caused by the root torsional spring mentioned earlier is $y=\frac{Fl^{\text{2}}}{K}$. From the relation $\frac{Fl^{\text{2}}}{K}=\frac{Fl^{\text{3}}}{3EI_{b}}$, the stiffness of the root torsional spring is obtained approximately as $K=\frac{3EI_{b}}{l}$.

A branch is a deformable body, and a tree consists of many such branches; a complete theoretical vibration analysis of a real tree is therefore practically impossible. If each fruit branch is simplified into a rigid-body-torsional-spring system, the computation is greatly reduced, and a theoretical vibration analysis becomes feasible. Setting the torsional spring stiffness of the simplified branch as $K=\frac{3EI_{b}}{l}$ will inevitably cause some deviation from the actual vibration behavior. Using a fruit branch fixed at one end and loaded at the other, we now evaluate the discrepancy between the real beam and the simplified model.

Assume that the fruit branch has a density ρ and cross-sectional area A, with a lumped mass $M_{e}$ attached at the tip. The branch is fixed at the root and free at the tip. Let the deflection of the branch be y(x,t). The bending vibration equation of a uniform beam is:


$$\begin{array}{c} EI_{b}\frac{\partial^{\text{4}}y}{\partial x^{\text{4}}}+\rho A\frac{\partial^{\text{2}}y}{\partial t^{\text{2}}}=0 \end{array}$$ (8)

The boundary conditions for a cantilever beam are: at the root x=0, y=0 and $y^{\prime }=0$; at the free end x=l, $y^{\prime \prime }=0$ and $EI_{b}y^{\prime \prime \prime }=M_{e}\ddot{y}$. Assuming a solution of the form y=ϕ(x)sinωt and substituting it into Eq. (8), and introducing the dimensionless parameters $\beta^{\text{4}}=\frac{\rho A\omega^{\text{2}}}{EI_{b}}l^{\text{4}}$, $\alpha =\frac{M_{e}}{\rho Al}$, the characteristic equation is obtained as:


$$\begin{array}{c} 1+cos\beta cosh\beta +\alpha \beta (sin\beta cosh\beta -cos\beta sinh\beta )=0 \end{array}$$ (9)

The first-order natural frequency corresponding to the vibration is:


$$\begin{array}{c} \omega_{\text{real}}=\beta_{\text{1}}^{\text{2}}\sqrt{\frac{EI_{b}}{\rho Al^{\text{4}}}} \end{array}$$ (10)

where $\beta_{\text{1}}$ is the smallest positive solution of Eq. (10). When there is no tip mass (α=0), the solution gives $\beta_{\text{1}}\approx 1.875$, $\beta_{\text{1}}^{\text{2}}\approx 3.516$.

In the simplified model, the fruit branch is regarded as a uniform rigid bar, and the elasticity of the branch is replaced by a torsional spring at the root with stiffness $K=\frac{3EI_{b}}{l}$. The mass of the rigid bar is m=ρAl, and its moment of inertia about the root is taken as $J_{\text{0}}=\frac{\text{1}}{\text{3}}ml^{\text{2}}$. The lumped mass at the tip of the rigid bar is $M_{e}$, its displacement is lθ, and the kinetic energy of the rigid-bar system is:


$$\begin{array}{c} T=\frac{\text{1}}{\text{2}}J_{\text{0}}{\dot{\theta }}^{\text{2}}+\frac{\text{1}}{\text{2}}M_{e}(l\dot{\theta }{)}^{\text{2}}=\frac{\text{1}}{\text{2}}\left(\frac{\text{1}}{\text{3}}m+M_{e}\right)l^{\text{2}}{\dot{\theta }}^{\text{2}} \end{array}$$ (11)

The potential energy of the system is provided by the torsional spring:


$$\begin{array}{c} V=\frac{\text{1}}{\text{2}}K\theta^{\text{2}} \end{array}$$ (12)

The kinetic equation for the fruit branch system is:


$$\begin{array}{c} \left(\frac{\text{1}}{\text{3}}\rho Al^{\text{3}}+M_{e}l^{\text{2}}\right)\ddot{\theta }\text{+}\frac{3EI_{b}}{l}\theta =0 \end{array}$$ (13)

the natural frequency of the fruit branch is:


$$\begin{array}{c} \omega_{s}=\sqrt{\frac{3EI_{b}}{l^{\text{3}}\left(M_{e}+\frac{\text{1}}{\text{3}}\rho Al\right)}} \end{array}$$ (14)

Taking $\alpha =\frac{M_{e}}{\rho Al}$, Eq. (14) becomes:


$$\begin{array}{c} \omega_{s}=\sqrt{\frac{\text{3}}{\alpha +1/3}}\sqrt{\frac{EI_{b}}{\rho Al^{\text{4}}}} \end{array}$$ (15)

The relative error in frequency is:


$$\begin{array}{c} \varepsilon =\frac{\omega_{\text{real}}-\omega_{s}}{\omega_{\text{real}}}=1-\frac{\omega_{s}}{\omega_{\text{real}}} \end{array}$$ (16)

Substituting Eqs and into, the error becomes a function of the mass ratio α:


$$\begin{array}{c} \varepsilon \left(\alpha \right)=1-\sqrt{\frac{\text{3}}{\beta_{\text{1}}^{\text{4}}\left(\alpha +1/3\right)}} \end{array}$$ (17)

When there is no concentrated mass at the branch tip α=0, the maximum relative error in the natural frequency is $\varepsilon \left(0\right)\approx 1-\frac{\text{3}}{3.516}\approx 14.7\%$. As the tip mass $M_{e}$ increases, the error decreases rapidly. When α≥0.6, the relative error ε≤5%; when α→∞, the relative error ε→0, which means that the fundamental frequency of the simplified model equals that of the actual model.

When the fruit branch is simplified to a combination of a uniform rigid bar and a torsional spring, the details of bending deformation are neglected, but the relative error in the fundamental frequency remains small. Since the tip of a blue honeysuckle branch in the field typically carries both branches and fruits, this simplified model is simple, effective, and sufficiently accurate.

By equating the fundamental natural frequency of the simplified model to that of the actual elastic fruit branch, an equivalent angular stiffness can be derived. Setting $\omega_{s}=\omega_{\text{real}}$, the angular stiffness K is obtained as:


$$\begin{array}{c} K=\omega_{\text{real}}^{\text{2}}\left(\frac{\text{1}}{\text{3}}\rho Al+M_{e}\right)l^{\text{2}}=\beta_{\text{1}}^{\text{4}}\frac{EI_{b}}{\rho Al^{\text{4}}}\left(\frac{\text{1}}{\text{3}}\rho Al+M_{e}\right)l^{\text{2}} \end{array}$$ (18)

This K is match the real beam in terms of frequency:


$$\begin{array}{c} K=\frac{EI_{b}}{l}\beta_{\text{1}}^{\text{4}}\left(\alpha +\frac{\text{1}}{\text{3}}\right)\; \end{array}$$ (19)

Dividing Eq. (19) by $\frac{3EI_{b}}{l}$ yields the stiffness correction factor:


$$\begin{array}{c} \eta \left(\alpha \right)=\frac{\beta_{\text{1}}^{\text{4}}}{\text{3}}\left(\alpha +\frac{\text{1}}{\text{3}}\right) \end{array}$$ (20)

where $\beta_{\text{1}}$ is a function of α, determined by Eq. (9). The variation of η with α can be obtained numerically. When α=0, then $\beta_{\text{1}}\approx 1.875$, and the maximum stiffness correction factor is η(0)≈1.374. To accurately reproduce the first-order frequency of a real branch, the angular stiffness can be amplified by a correction factor that depending on the mass ratio between the applied load and the branch mass α. Since the branches of field blue honeysuckle generally carry fruits or sub-branches whose mass exceeds that of the branch itself, this study adopts the statically equivalent stiffness $K=\frac{3EI_{b}}{l}$.

Assuming that the fruit branch cross section is circular with diameter d, then $K=\frac{\text{3}E\pi d^{\text{4}}}{\text{64}l}$. If the density of the fruit branch is ρ, the mass of the fruit branch can be expressed as $m=\frac{\pi d^{\text{2}}\rho l}{\text{4}}$. Consequently, the elastic constant of the torsional spring is obtained as:


$$\begin{array}{c} K=\frac{\text{3}Em^{\text{2}}}{4\pi \rho^{\text{2}}l^{\text{3}}}=G\frac{m^{\text{2}}}{l^{\text{3}}} \end{array}$$ (21)

where $G=\frac{\text{3}E}{4\pi \rho^{\text{2}}}$. Let the elastic constant of the torsional spring of the main stem be $k_{\text{1}}=K=G\frac{m^{\text{2}}}{l^{\text{3}}}$. Based on the diameters ratio, the elastic constant of the upper stem is $k_{\text{2}}=\frac{a_{\text{2}}^{\text{2}}}{b_{\text{2}}^{\text{3}}}k_{\text{1}}$ and that of the side branch is $k_{\text{3}}=\frac{a_{\text{3}}^{\text{2}}}{b_{\text{3}}^{\text{3}}}k_{\text{1}}$. Introducing the parameters $\omega_{\text{0}}^{\text{2}}=\frac{Gm_{\text{1}}}{l_{\text{1}}^{\text{5}}}=\frac{\text{3}Em_{\text{1}}}{4\pi \rho^{\text{2}}l_{\text{1}}^{\text{5}}}$, $G_{\text{0}}=\frac{g}{l_{\text{1}}}$, $d_{i}=\frac{c_{i}}{m_{\text{1}}l_{\text{1}}^{\text{2}}}$ (i=1,2,3), $N\left(t\right)=-\frac{A\Omega^{\text{2}}}{l_{\text{1}}}cos\left(\Omega t\right)$, Eq. (7) can be rewritten as:


$$\begin{array}{c} M\ddot{q}+C\dot{q}+\left[K_{\text{0}}+N\left(t\right)D\right]q=F\left(t\right) \end{array}$$ (22)

where $M=\left[\begin{array}{ccc} @ccc@A_{11} & A_{12} & A_{13}\\ A_{12} & A_{22} & 0\\ A_{13} & 0 & A_{33} \end{array}\right]$, $C=\left[\begin{array}{ccc} @ccc@d_{\text{1}} & 0 & 0\\ 0 & d_{\text{2}} & 0\\ 0 & 0 & d_{\text{3}} \end{array}\right]$, $K_{\text{0}}=\left[\begin{array}{ccc} @ccc@K_{11} & K_{12} & 0\\ K_{12} & K_{22} & 0\\ 0 & 0 & K_{33} \end{array}\right]$, $D=\frac{a_{\text{3}}b_{\text{3}}}{\text{2}}\left[\begin{array}{ccc} @ccc@1 & 0 & 1\\ 0 & 0 & 0\\ 1 & 0 & 1 \end{array}\right]$, $F\left(t\right)=\left[\begin{array}{c} @c@\begin{array}{c} B_{\text{1}}N\left(t\right)+\frac{a_{\text{3}}b_{\text{3}}}{\text{2}}G_{\text{0}}\\ \frac{a_{\text{2}}b_{\text{2}}}{\text{2}}N\left(t\right)\\ \frac{a_{\text{3}}b_{\text{3}}}{\text{2}}G_{\text{0}} \end{array} \end{array}\right]$, $A_{11}=\frac{\text{1}}{\text{3}}+a_{\text{2}}\;\left(1-b_{\text{2}}+\frac{b_{\text{2}}^{\text{2}}}{\text{3}}\right)+a_{\text{3}}\;\left(1+\frac{b_{\text{3}}^{\text{2}}}{\text{3}}\right)$, $A_{12}=a_{\text{2}}\;\left(\frac{b_{\text{2}}^{\text{2}}}{\text{3}}-\frac{b_{\text{2}}}{\text{2}}\right)$, $A_{13}=A_{33}=\frac{a_{\text{3}}b_{\text{3}}^{\text{2}}}{\text{3}}$, $A_{22}=\frac{a_{\text{2}}b_{\text{2}}^{\text{2}}}{\text{3}}$, $K_{11}=\omega_{\text{0}}^{\text{2}}-B_{\text{1}}G_{\text{0}}$, $K_{12}=-\frac{a_{\text{2}}b_{\text{2}}}{\text{2}}G_{\text{0}}$, $K_{22}=\frac{a_{\text{2}}^{\text{2}}}{b_{\text{2}}^{\text{3}}}\omega_{\text{0}}^{\text{2}}-\frac{a_{\text{2}}b_{\text{2}}}{\text{2}}G_{\text{0}}$, $K_{33}=\frac{a_{\text{3}}^{\text{2}}}{b_{\text{3}}^{\text{3}}}\omega_{\text{0}}^{\text{2}}$, $B_{\text{1}}=\frac{\text{1}}{\text{2}}+a_{\text{2}}+a_{\text{3}}+\frac{a_{\text{2}}b_{\text{2}}}{\text{2}}$.

#### 2.2.4 Stability of the dynamic equations.

If the constant stiffness matrix $K_{\text{0}}$ in Eq. (22) is not positive definite, the equations have no real solution; physically, the fruit branch system topples under gravity and exhibits static instability. To study the vibration characteristics of the fruit branch system, it is assumed that the system is in a statically stable state. Furthermore, the term F(t) does not affect the stability of the system; therefore, only the homogeneous equation is considered:


$$\begin{array}{c} M\ddot{q}+C\dot{q}+\left[K_{\text{0}}+N\left(t\right)D\right]q=0 \end{array}$$ (23)

The system modes are obtained by setting the excitation term N(t)=0, and performing a modal coordinate transformation. According to Eq. (23), the eigenvalue equation of the system can be written as:


$$\begin{array}{c} \left(K_{\text{0}}-\omega_{i}^{\text{2}}M\right)\phi_{i}=0 \end{array}$$ (24)

By solving Eq. (24), the natural frequencies of the system $\omega_{\text{1}},\omega_{\text{2}},\omega_{\text{3}}$ and the mass‑normalized mode shape matrix $\Phi =\left[\phi_{\text{1}},\phi_{\text{2}},\phi_{\text{3}}\right]$ are obtained, where $\phi_{\text{1}},\phi_{\text{2}},\phi_{\text{3}}$ are the corresponding three eigenvectors that satisfy the orthogonality conditions $\Phi^{T}M\Phi =I$ and $\Phi^{T}K_{\text{0}}\Phi =diag\left(\omega_{\text{1}}^{\text{2}},\omega_{\text{2}}^{\text{2}},\omega_{\text{3}}^{\text{2}}\right)$. Using the modal coordinates transformation q=Φx, substituting into the homogeneous Eq. (23), and left‑multiplying by $\Phi^{T}$, one obtains:


$$\begin{array}{c} \ddot{x}+\Phi^{T}C\Phi \dot{x}+\left[\Lambda +N\left(t\right)\Gamma \right]x=0 \end{array}$$ (25)

where $\Lambda =diag\left(\omega_{\text{1}}^{\text{2}},\omega_{\text{2}}^{\text{2}},\omega_{\text{3}}^{\text{2}}\right)$, $\Gamma =\Phi^{T}D\Phi$.

Under weak damping, if the off‑diagonal elements of $\Phi^{T}C\Phi$ are neglected and only its diagonal entries are retained as $2\zeta_{i}\omega_{i}=\phi_{i}^{T}C\phi_{i}$, and the off‑diagonal elements of the parametric resonance matrix Γ, which represent the dynamic coupling coefficients between different vibration modes, induced by external periodic excitation, are also neglected, Eq. (25) can be written as three independent equations:


$$\begin{array}{c} {\ddot{x}}_{i}+2\zeta_{i}\omega_{i}{\dot{x}}_{i}+\left[\omega_{i}^{\text{2}}-\frac{A\Omega^{\text{2}}}{l_{\text{1}}}\gamma_{ii}cos\left(\Omega t\right)\right]x_{i}=0,\;i=1,2,3 \end{array}$$ (26)

where $\gamma_{ii}=\phi_{i}^{T}D\phi_{i}$, i=1,2,3 denotes the parametric excitation coefficient. Introducing the dimensionless time τ=Ωt, Eq. (26) becomes:


$$\begin{array}{c} {x^{\prime \prime }}_{i}+\frac{2\zeta_{i}\omega_{i}}{\Omega }{x^{\prime }}_{i}+\left(\frac{\omega_{i}^{\text{2}}}{\Omega^{\text{2}}}-\frac{A\gamma_{ii}}{l_{\text{1}}}cos\tau \right)x_{i}=0,\;i=1,2,3 \end{array}$$ (27)

where ′=d/dτ. The standard form of the Mathieu equation is usually written as $\ddot{x}+2\beta \dot{x}+(\delta +\varepsilon cost)x=0$. Comparing Eq. (27) with this standard form, the coefficient values for each equation are: $\delta_{i}=\omega_{i}^{\text{2}}/\Omega^{\text{2}}$, $\varepsilon_{i}=A\gamma_{ii}/l_{\text{1}}$, $\beta_{i}=\zeta_{i}\omega_{i}/\Omega$, i=1,2,3. Because the sign of the cosine term can be changed to positive by a time shift, the stability boundaries of Eq. (27) are identical to those of the standard Mathieu equation. The instability regions of the Mathieu equation occur near $\delta \approx n^{\text{2}}/4$ (n=0,1,2,…), with the principal resonance at n=1 and the secondary resonance at n=2. Since the width of the secondary resonance instability region is much narrower than that of the principal resonance region, and the secondary resonance is more sensitive to damping, this study focuses on the principal resonance region. The range of the principal resonance is:


$$\begin{array}{c} \left|\delta -\frac{\text{1}}{\text{4}}\right|<\frac{\text{1}}{\text{2}}\sqrt{\varepsilon^{\text{2}}-\text{4}\beta^{\text{2}}} \end{array}$$ (28)

Substituting the coefficient values$\delta_{i}=\omega_{i}^{\text{2}}/\Omega^{\text{2}}$, $\varepsilon_{i}=A\gamma_{ii}/l_{\text{1}}$, $\beta_{i}=\zeta_{i}\omega_{i}/\Omega$ into Eq. (28) yields the condition for the system to enter the instability region. The relationship between the minimum excitation amplitude and the excitation frequency is:


$$\begin{array}{c} \left|A\right|=\frac{l_{\text{1}}}{2\left|\gamma_{ii}\right|}\sqrt{{\left(\frac{4\omega_{i}^{\text{2}}}{\Omega^{\text{2}}}-1\right)}^{\text{2}}+{\left(\frac{4\zeta_{i}\omega_{i}}{\Omega }\right)}^{\text{2}}} \end{array}$$ (29)

For an undamped system, the minimum amplitude A required to cause instability satisfies:


$$\begin{array}{c} \left|A\right|=\frac{\left|4\omega_{i}^{\text{2}}-\Omega^{\text{2}}\right|l_{\text{1}}}{2\Omega^{\text{2}}\left|\gamma_{ii}\right|} \end{array}$$ (30)

Eq. (28) also reveals the width of the instability region. According to Eq. (30), the boundaries of the region satisfy:


$$\begin{array}{c} \Omega^{\text{2}}=4\omega_{i}^{\text{2}}\mp 2\frac{A\Omega^{\text{2}}}{l_{\text{1}}}\left|\gamma_{ii}\right| \end{array}$$ (31)

When $\frac{A\Omega^{\text{2}}}{l_{\text{1}}}$ is small, Eq. (31) can be approximated by the expansion: $\Omega \approx 2\omega_{i}\left(1\mp \frac{\left|\gamma_{ii}\right|}{4\omega_{i}^{\text{2}}}\frac{A\Omega^{\text{2}}}{l_{\text{1}}}\right)$. Thus, the width of the instability region ΔΩ can be expressed as:


$$\begin{array}{c} \Delta \Omega =\Omega_{\text{max}}-\Omega_{\text{min}}\approx \frac{\left|\gamma_{ii}\right|}{2\omega_{i}}\frac{A\Omega^{\text{2}}}{l_{\text{1}}}. \end{array}$$ (32)

Based on the characteristics of the principal resonance region of an undamped system $\omega_{i}^{\text{2}}/\Omega^{\text{2}}=\frac{\text{1}}{\text{4}}$,


$$\begin{array}{c} \Delta \Omega \approx \frac{\text{1}}{2\omega_{i}}\cdot \frac{4A\omega_{i}^{\text{2}}}{l_{\text{1}}}\cdot \left|\gamma_{ii}\right|=\frac{2A\omega_{i}\left|\gamma_{ii}\right|}{l_{\text{1}}} \end{array}$$ (33)

the width of the instability region is proportional to the excitation amplitude A, with the proportionality coefficient equal to $\frac{2\omega_{i}\left|\gamma_{ii}\right|}{l_{\text{1}}}$. It also can be seen that a smaller $l_{\text{1}}$ leads to a wider instability region, making the system more prone to instability. Conversely, a larger $l_{\text{1}}$ makes the system more stable.

Parametric resonance (Ω≈2ω) and ordinary resonance (Ω≈ω) both manifests as large-amplitude vibrations of a system under specific excitation frequencies; nevertheless, their underlying physical mechanisms are fundamentally different. Ordinary resonance is a linear forced response. The external excitation acts as an inhomogeneous term applied directly to the system. The general equation of motion is given by:


$$\begin{array}{c} \ddot{x}+2\zeta \omega \dot{x}+\omega^{\text{2}}x=F_{\text{0}}cos\left(\Omega t\right) \end{array}$$ (34)

where x is the displacement, ω is the undamped natural angular frequency, ζ is the damping ratio, $F_{\text{0}}$ is the amplitude of the external force, Ω is the angular frequency of the excitation, and t is time. When the excitation frequency Ω≈ω, the amplitude is linearly amplified. The response amplitude is proportional to the excitation amplitude $F_{\text{0}}$. The amplification factor is governed by damping, and an increase in damping causes the resonance peak to drop sharply.

Parametric resonance, in contrast, is a parametric instability phenomenon. The excitation does not provide a direct external force but instead periodically modulates the stiffness of the system. It is described by a homogeneous differential equation with time-varying coefficients, typically of the form:


$$\begin{array}{c} \ddot{x}+\omega^{\text{2}}\left[1+\varepsilon cos\text{\textasciitilde{}(}\Omega t\text{)}\right]x=0 \end{array}$$ (35)

where ω is the natural angular frequency of the unmodulated system when ε=0, ε is the parametric modulation depth. Ω is the angular frequency of the modulation. When the modulation frequency satisfies Ω≈2ω, the system undergoes severe dynamic instability. When the modulation depth ε exceeds a critical value determined by the damping, the vibration amplitude grows exponentially until nonlinear effects limit it. This behavior is entirely different from linear forced resonance, where the amplitude is proportional to the excitation amplitude.

In vibratory harvesting of blue honeysuckle, applying an excitation near the natural frequency of the branch with a large amplitude forcibly drives the branch. This approach requires high energy and can easily cause bark abrasion or branch breakage. If parametric resonance is employed, only a small-amplitude excitation at approximately twice the natural frequency of the branch needs to be applied. This excitation periodically alters the effective stiffness of the branch. Once the modulation magnitude exceeds the instability threshold, the branch develops intense transverse oscillations.

#### 2.2.5 Stability of the dynamic equation considering only the side branch.

Under the assumption of weak inertial coupling of the side branch with the main stem and the upper stem, by setting the off-diagonal elements of the mass matrix $A_{12}$, $A_{13}$ to zero and simultaneously neglecting the off-diagonal elements of the parameter matrix Γ, the stability can be analyzed solely based on the dynamic equation of the side branch. Under these conditions, the third equation in Eq. (23) reduces to:


$$\begin{array}{c} \frac{b_{\text{3}}^{\text{2}}}{\text{3}}{\ddot{q}}_{\text{3}}+\frac{d_{\text{3}}}{a_{\text{3}}}{\dot{q}}_{\text{3}}+\left(\frac{a_{\text{3}}}{b_{\text{3}}^{\text{3}}}\omega_{\text{0}}^{\text{2}}-\frac{b_{\text{3}}}{\text{2}}\frac{A\Omega^{\text{2}}}{l_{\text{1}}}cos\left(\Omega t\right)\right)q_{\text{3}}=0, \end{array}$$ (36)

where $d_{\text{3}}=\frac{c_{\text{3}}}{m_{\text{1}}l_{\text{1}}^{\text{2}}}$. By further introducing τ=Ωt, Eq. (36) becomes the standard form of the Mathieu equation:


$$\begin{array}{c} {q^{\prime \prime }}_{\text{3}}+2\overset{―}{\beta }{q^{\prime \prime }}_{\text{3}}+(\overset{―}{\delta }-\overset{―}{\varepsilon }cos\tau )q_{\text{3}}=0, \end{array}$$ (37)

where $\overset{―}{\delta }=\frac{3a_{\text{3}}\omega_{\text{0}}^{\text{2}}}{b_{\text{3}}^{\text{5}}\Omega^{\text{2}}}$, $\overset{―}{\varepsilon }=\frac{3A}{2b_{\text{3}}l_{\text{1}}}$, $\overset{―}{\beta }=\frac{3d_{\text{3}}}{2a_{\text{3}}b_{\text{3}}^{\text{2}}\Omega }$.

The instability region of the side branch appears at $\overset{―}{\delta }=\frac{\text{1}}{\text{4}}$, which corresponds to the principal resonance region of the Mathieu equation. In this region, the excitation frequency Ω is twice the lowest-order natural frequency of the side branch. Based on this resonance condition, one obtains:


$$\begin{array}{c} \frac{a_{\text{3}}}{b_{\text{3}}^{\text{5}}}=\frac{\Omega^{\text{2}}}{12\omega_{\text{0}}^{\text{2}}} \end{array}$$ (38)

Expressed further in terms of the geometric parameters of the side branch, Eq. (38) becomes:


$$\begin{array}{c} \Omega^{\text{2}}=\frac{\text{9}Ed_{\text{3}}^{\text{2}}}{4\rho l_{\text{3}}^{\text{4}}} \end{array}$$ (39)

Therefore, when considering the vibration of the side branch in isolation, if the geometric parameters of the side branch satisfy Eq. (39), the branch system is unstable. From Eq. (39), it can be seen that a larger diameter of the side branch corresponds to a higher excitation frequency at which instability occurs, whereas a longer side branch leads to a lower excitation frequency for instability.

#### 2.2.6 Vibration of the fruit branch system based on numerical analysis.

Because the damping of the fruit branch structure is generally small, its influence is neglected in the following numerical analysis. Based on the branch data in Table 1, the main stem has a length of 150 mm and a diameter of 6 mm. The upper stem has a length of 50 mm long and a diameter of 6 mm. The side branch has a length of 156 mm and a diameter of 2.15 mm. From these data, the mass and length ratios are calculated as 0.33 for the upper stem, and 0.137 and 1.04 for the side branch, respectively. All these values above are substituted into Eq. (30). Taking the harvesting excitation frequency as the independent variable, the instability region boundaries of the blue honeysuckle branch under principal resonance are solved using Mathematica. Furthermore, by varying the parameters in Eq. (23), the influence of changes in the geometric parameters of the fruit branch on the instability region is systematically analyzed. Using the branch length ratio as the independent variable and following Eq. (39), the relationship between the geometric parameters of the side branch and the excitation frequency at which instability occurs is calculated.

A finite element model of the Y-shaped fruit branch is established, and the physical properties of the blue honeysuckle branch listed in Table 1 are assigned to the model. The natural frequencies of the fruit branch system are computed using ABAQUS, and an explicit dynamics analysis is performed using ABAQUS to determine the maximum vertical response amplitude of the side branch under different excitation frequencies.

## 3. Results

### 3.1 Instability regions of the fruit branch system

#### 3.1.1 Variation of the instability regions with the fruit branch parameters.

Based on the measured data of the blue honeysuckle branch presented in Table 1, numerical calculations are performed using Mathematica to determine the instability regions of the fruit branch system related to excitation frequencies and amplitudes. According to Eq. (24), the simplified branch system possesses three modes, therefore, Eq. (30) yields three corresponding instability regions. The first-order mode corresponds to the lowest natural frequency and mode shape of the branch system. In practice, the low‑frequency mode is more easily excited; consequently, subsequent numerical calculations consider only the first-order mode of the branch system.

**Fig 3:**
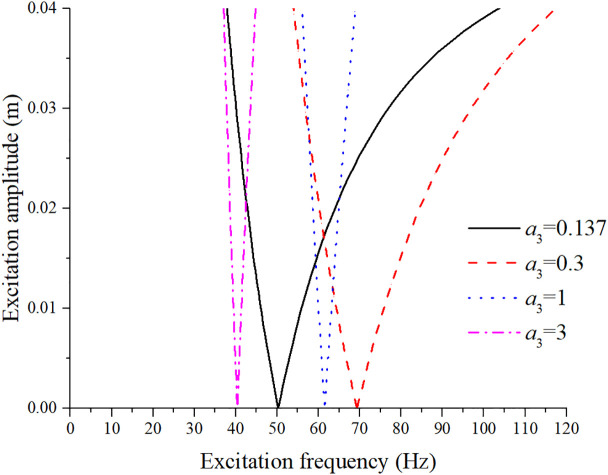
Variation of the instability region with *a*_3_.

In the model, the main stem and upper stem share the same diameter and are arranged coaxially, with the side branch perpendicular to the main stem. The three natural frequencies of the branch system computed from Eq. (24) are 25.16 Hz, 52.12 Hz, and 771.45 Hz. When only the mass ratio of the side branch to the main stem $a_{\text{3}}$ varies among the above parameters, the variation of the instability regions for the first order mode of the branch system, as obtained from Eq. (30), is shown in Fig 3.

As shown in Fig 3, under the same excitation amplitude, as $a_{\text{3}}$ gradually increases, the instability region of the fruit branch system first shifts toward the high-frequency range and then moves to the low-frequency range. This phenomenon can be attributed to Eq. (23): the equivalent stiffness of the side branch is proportional to $K_{33}=\frac{a_{\text{3}}^{\text{2}}}{b_{\text{3}}^{\text{3}}}\omega_{\text{0}}^{\text{2}}$. Therefore, as $a_{\text{3}}$ increases, the equivalent stiffness grows, raising the natural frequency of the side branch and consequently increasing the first-order natural frequency of the fruit branch system. However, the increase in side branch mass also increases the total inertia of the system, i.e., the terms $A_{11}$, $A_{13}$ rise. When $a_{\text{3}}$ exceeds a certain critical value, the inertial effect gradually becomes dominant, causing the natural frequency of the entire system to decrease, and the instability region to shift toward the low-frequency direction.

According to Eq. (33), under constant excitation amplitude, the width of the instability region is determined by the product of the modal natural frequency $\omega_{i}$ and the coupling coefficient $\gamma_{ii}$. For the first-order mode, during the initial increase of $a_{\text{3}}$, both $\omega_{\text{1}}$ and $\gamma_{11}$ increase rapidly, so their product increases and the instability region widens. In the later stage, $\omega_{\text{1}}$ decreases and the growth rate of $\gamma_{11}$ slows down or decreases, causing the product to diminish and the region to narrow. This results in the width of the instability region first increasing and then decreasing.

As shown in Fig 3, to make the instability regions corresponding to different excitation frequencies overlap, the excitation amplitude must be very large. However, such high‑frequency, high‑amplitude vibration conditions can easily cause damage to the fruit branch.

**Fig 4:**
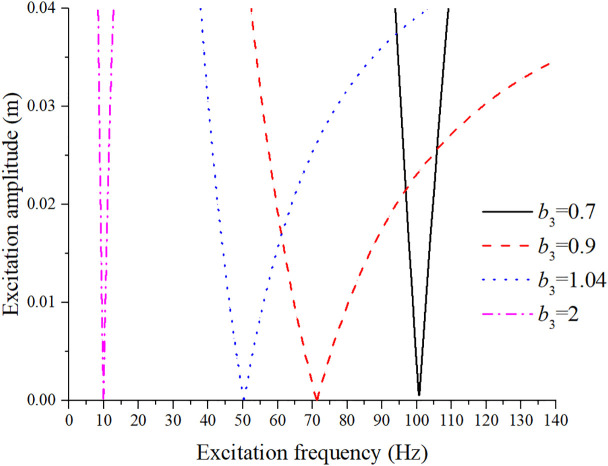
Variation of the instability region with *b*_3_.

When only the length ratio of the side branch to the main stem $b_{\text{3}}$ is varied, the calculated instability regions are shown in Fig 4.

As shown in Fig 4, when $b_{\text{3}}$ increases, under the same excitation amplitude, the instability regions of the fruit branch system continuously shift towards the low-frequency range. The reason is attributed to Eq. (23): the equivalent stiffness of the side branch $K_{33}=\frac{a_{\text{3}}^{\text{2}}}{b_{\text{3}}^{\text{3}}}\omega_{\text{0}}^{\text{2}}$ decreases rapidly, while the terms $A_{11}$, $A_{13}$ in the mass matrix increase. These factors lead to a reduction in the first-order modal frequency of the fruit branch system. The rapid shift of the unstable frequency toward the low-frequency region indicates that a long side branch easily induces vibrational instability in the fruit branch system.

**Fig 5:**
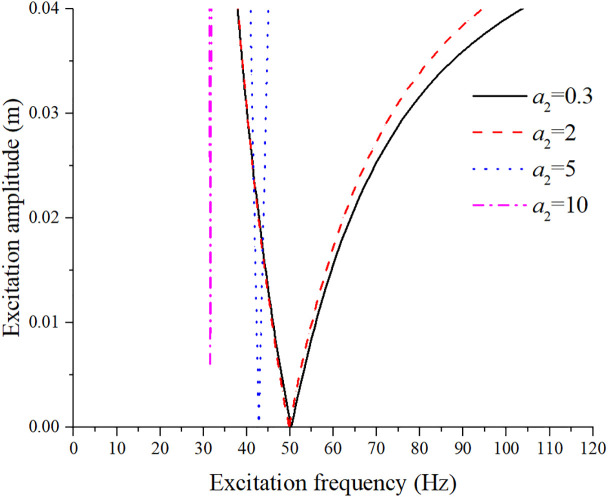
Variation of the instability region with *a*_2_.

When only the mass ratio of the upper stem to the main stem $a_{\text{2}}$ is varied, the corresponding instability regions are shown in Fig 5.

Fig 5 shows that, when the mass of the upper stem increases, the instability region of the fruit branch system gradually shifts toward the low-frequency range. According to Eq. (23), the terms in the mass matrix directly related to $a_{\text{2}}$ are $A_{11}$, $A_{12}$, and $A_{22}$, which all increase linearly with $a_{\text{2}}$. This implies that an increase in the mass of the upper stem directly raises the total moment of inertia of the system, thereby shifting the instability region toward the low-frequency range. When the value of $a_{\text{2}}$ exceeds 5, the width of the instability region decreases significantly, in fact, for small excitation amplitudes, it tends to disappear.

**Fig 6:**
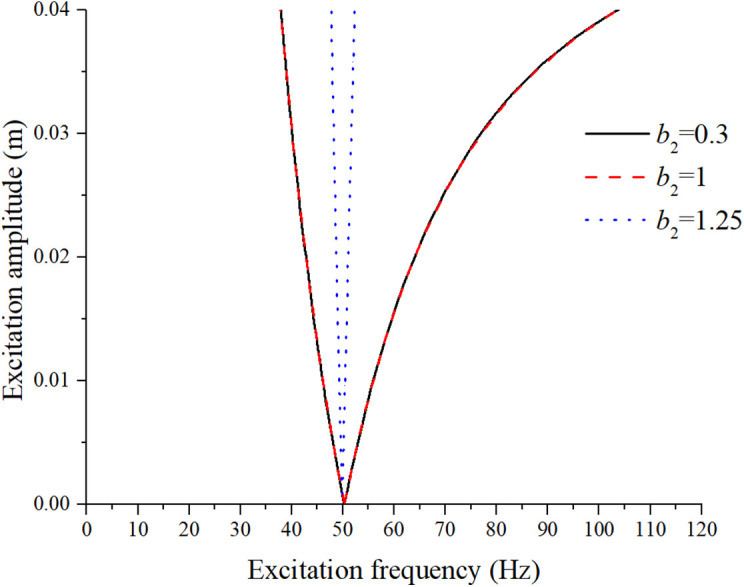
Variation of the instability region with *b*_2_.

When only the length ratio of the upper stem to the main stem $b_{\text{2}}$ is varied, the vibration instability regions are shown in Fig 6.

In the numerical calculation, the upper stem is short and light, and it moves almost together with the main stem, exhibiting a small variation in its rotation angle relative to the main stem. Consequently, the first-order vibration mode is primarily governed by the main stem and the side branch, and $b_{\text{2}}$ has little effect on the location of the instability region. As shown in Fig 6, when the length of the upper stem is comparable to the main stem itself, an increase in $b_{\text{2}}$ leads to a significant narrowing of the instability region, even causing this instability region to disappear completely.

#### 3.1.2 Considering vibration of the side branch in isolation.

**Fig 7:**
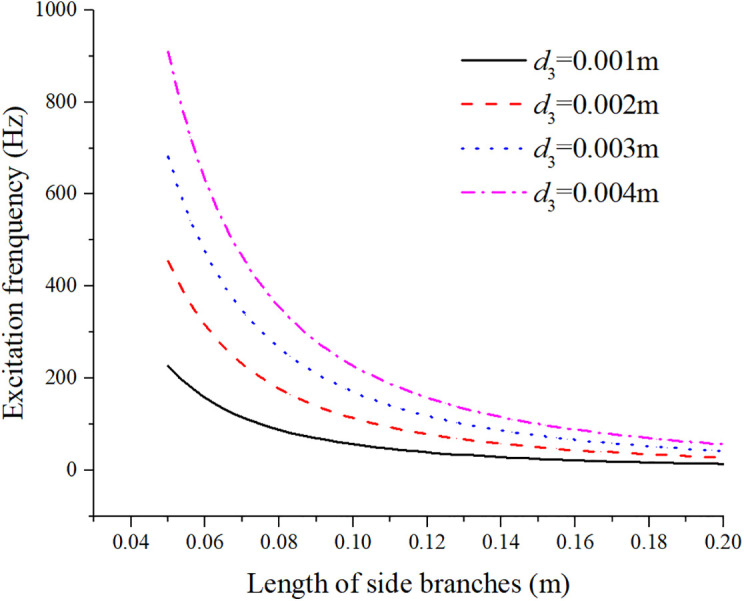
Geometric parameters of side branches for vibration instability.

If only the dynamic equations of the side branch are considered, the relationship among the parameters that leads to system instability must satisfy Eq. (39). The relationship between the geometric parameters of the side branch and the excitation frequency is shown in Fig 7.

As shown in Fig 7, for a fixed side branch length, a smaller branch diameter corresponds to a lower critical excitation frequency at which the branch experiences vibration instability. When the side branch is short, the excitation frequency that causes instability increases sharply with increasing diameter. If the diameter of the side branch is kept constant, a longer branch length leads to a lower excitation frequency for instability. Moreover, for larger diameters, the frequency that causes side branch instability decreases more rapidly as the branch length increases.

#### 3.1.3 Effect of branch length variation on system instability region.

Some parameters of the fruit branches are correlated. For example, when a branch is shortened, both its length and its mass change simultaneously. When a side branch is truncated, its length decreases from its initial value, and its mass decreases from its initial value. The evolution of the instability region after branch length change is therefore examined.

**Fig 8:**
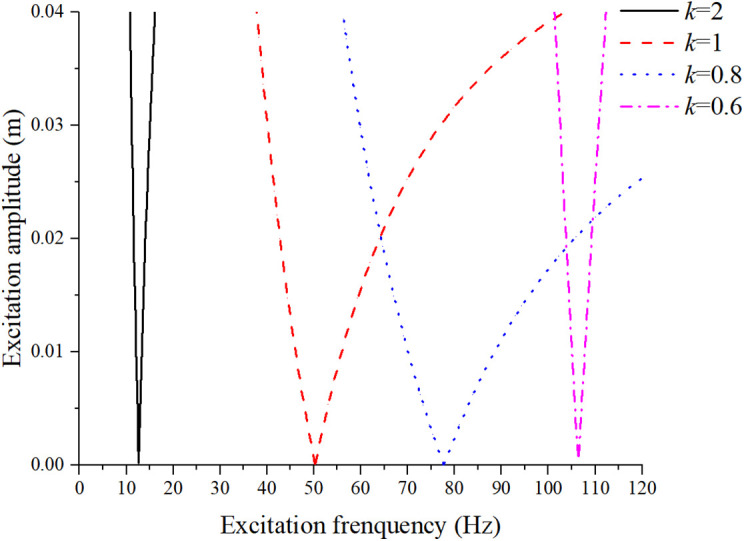
The instability region varies with the length of the side branch.

Assume the initial length of the branch is $l_{31}$, the varied length is $l_{32}$, the initial mass of the branch is $m_{31}=\frac{\rho \pi d_{\text{3}}^{\text{2}}}{\text{4}}l_{31}$, and the varied mass is $m_{32}=\frac{\rho \pi d_{\text{3}}^{\text{2}}}{\text{4}}l_{32}$. Consequently, we have $\frac{m_{31}}{m_{3\text{2}}}=\frac{l_{31}}{l_{3\text{2}}}$. Substituting these into the mass ratio, $m_{\text{31}}=a_{\text{31}}m$, $m_{3\text{2}}=a_{3\text{2}}m$, and the length ratio, $l_{\text{31}}=b_{\text{31}}l$, $l_{3\text{2}}=b_{3\text{2}}l$, one obtains:


$$\begin{array}{c} \frac{a_{31}}{a_{3\text{2}}}=\frac{b_{31}}{b_{3\text{2}}}=k \end{array}$$ (40)

where k can be treated as the ratio of the shortened length (or mass) to the initial length (or mass) of the branch. Assuming that when the length of the side branch varies, the vibration instability regions calculated from Eq. (30) are presented in Fig 8.

As shown in Fig 8, when the ratio of the length of the side branch to its initial length k becomes large, the excitation frequency of the instability region shifts toward lower frequencies, and the width of this instability region decreases significantly. When this ratio exceeds 2, with the main stem length fixed at 150 mm and the side branch length greater than 312 mm, the excitation frequency of the instability region is below 15 Hz. Conversely, when this ratio is less than 0.6, the excitation frequency of the instability region exceeds 105 Hz.

The trend in Fig 8 resembles that in Fig 4, with minor difference. However, it differs significantly from Fig 3, indicating that among the effects of parameters on the instability region, the side branch length plays a dominant role.

**Fig 9:**
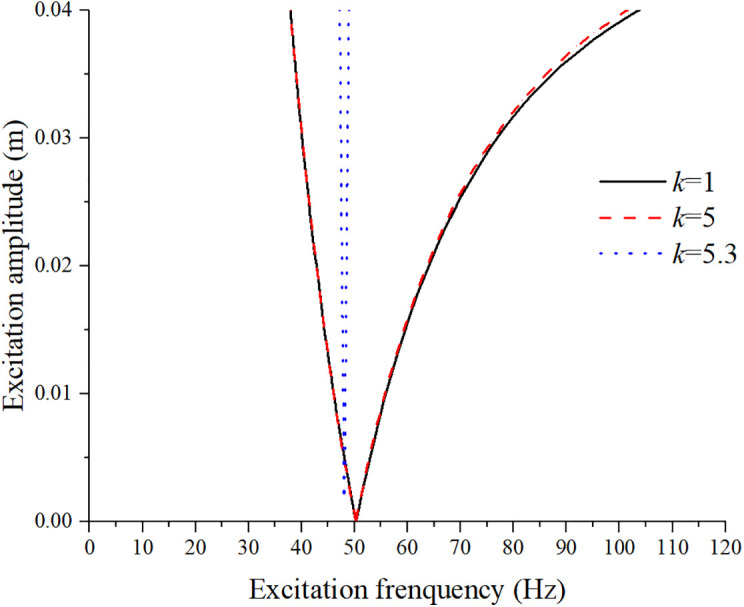
The instability region varies with the length of the upper stem.

Assuming that the upper stem length in the branch system varies, the vibration instability regions calculated from Eq. (30) are shown in Fig 9.

As shown in Fig 9, when the ratio of the upper stem length to its initial length k is less than 1, the instability region remains essentially unchanged. When the ratio is large, the excitation frequency corresponding to the instability region varies very little, while the width of this instability region decreases rapidly. This implies that when the upper stem length increases to 5.3 times its initial value (i.e., the upper stem length reaches 265 mm), the instability region almost completely disappears under small excitation amplitudes.

The trend in Fig 9 is essentially the same as that in Fig 6, which also indicates that the upper stem length has an important influence on the instability region.

### 3.2 Finite element analysis of branch system vibration

To verify the theoretical analysis results, ABAQUS is employed to simulate the vibration of the fruit branch system. A deformed branch system model is established. The geometric and material parameters of the fruit branch used in the simulation are kept consistent with those in the theoretical calculation. In the Assembly module, the side branch, the main stem, and the upper stem are merged to form a complete assembly. C3D10 elements are then used.

Two analysis steps are created: a frequency step and a steady-state dynamic step; the latter has an analysis time of 1 s. In the Load module, gravity is applied to the entire structure, and the boundary condition is set as a fixed root, thereby allowing the natural frequencies and mode shapes of the system to be computed. Subsequently, a displacement amplitude is prescribed at the root, with excitation frequencies of 5, 10, 15, 20, 25, 27.757, 30, 35, 40, 45, 50, 55, 55.514, 60, and 65 Hz. The maximum vertical displacement at the tip of the side branch is recorded for each excitation frequency.

#### 3.2.1 Modal analysis.

**Fig 10:**
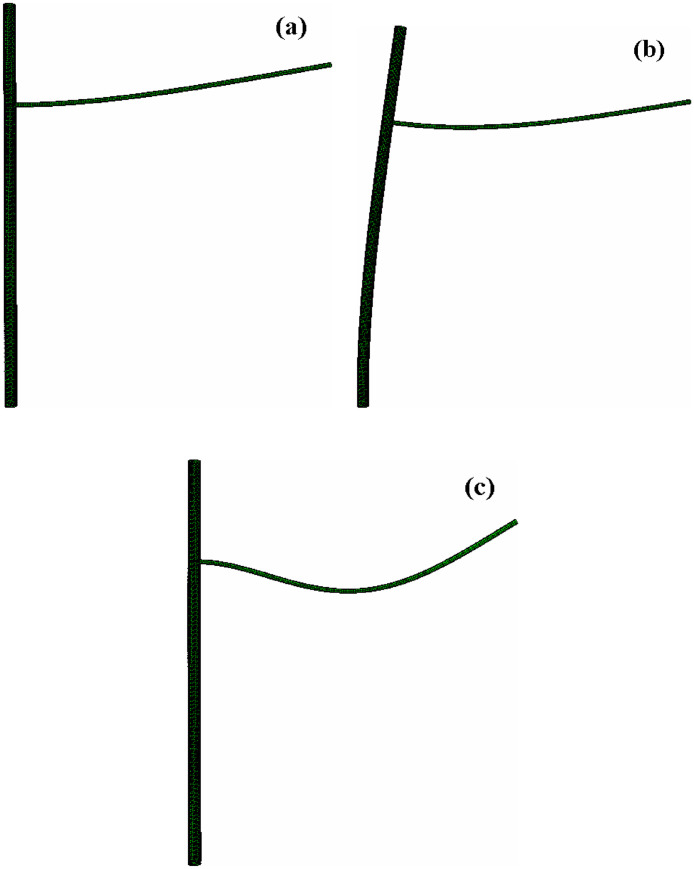
The first three mode shapes of the fruit branch system. (a) 27.757 Hz (b) 47.548 Hz (c) 178.29 Hz.

The elastic modulus and density of the main stem and side branch are assigned the same values as those used in the theoretical calculation, as shown in Table 1. Given that the damping of fruit branches is small, it is neglected in the calculation. Boundary constraints are applied by fixing the base of the main stem, and the vibration of the fruit branch is restricted to a single plane. The first three natural frequencies are 27.757 Hz, 47.548 Hz, and 178.29 Hz, respectively. Compared with the theoretical values 25.16 Hz, 52.12 Hz, and 771.45 Hz, the first two natural frequencies agree well. The third natural frequency shows a significant difference, which is attributed to the fact that the theoretical model is a simplified rigid body model that does not account for the bending mode of the fruit branch. The rigid body model can only describe the rigid‑body rotation of the fruit branch, whereas the finite element model incorporates the bending deformation of the branch. The first three mode shapes (with deformation magnified by a factor of 20) obtained from the finite element analysis are shown in Fig 10.

As shown in Fig 10, the mode shape at the natural frequency of 27.757 Hz is almost dominated by the vibration of the side branch. This is because under weak coupling conditions, when the side branch is lightweight and slender, the lowest-order mode of the system approaches the independent vibration mode of the side branch. Similarly, the mode shape at 178.29 Hz exhibits a bending mode of the side branch. However, this mode shape is not present in theoretical predictions. Consequently, none of the theoretically calculated natural frequencies are close to this value. In addition, because the main branch and the upper stem are modeled as an integrated entity in the finite element model, the natural frequency of the rotational motion of the upper stem is also absent in the finite element analysis.

#### 3.2.2 Explicit dynamics simulation of the fruit branch system.

**Fig 11:**
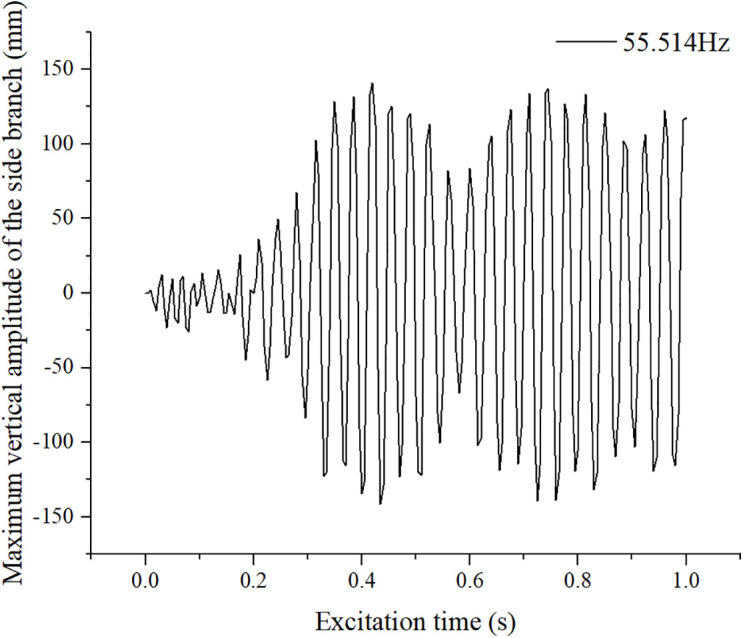
Maximum vertical amplitude of the side branch at an excitation frequency of 55.514 Hz.

Using the natural frequencies obtained from modal analysis, the vibration response of the fruit branch system under harmonic excitation at a frequency equal to twice the natural frequency is calculated. A displacement load with an amplitude of 20 mm is applied at the base of the main stem, and the excitation frequency is 55.514 Hz. Under this condition, the maximum amplitude of the side branch in the vertical direction is shown in Fig 11.

As shown in Fig 11, the simulation results are consistent with the theoretical analysis. When the excitation frequency is exactly twice the natural frequency, the vibration amplitude of the fruit branch increases exponentially, and the vibration frequency of the side branch approaches its natural frequency. This is a typical characteristic of parametric resonance. As time increases, the vibration amplitude of the fruit branch gradually decreases after reaching a peak. This is because, when the amplitude is small, the excitation frequency is exactly twice the first-order natural frequency, and the system enters a state of parametric resonance. Energy is rapidly injected into the system, causing the amplitude to grow exponentially. As the amplitude increases, the stiffness of the side branch changes due to large geometric deformation, causing the actual natural frequency of the fruit branch to deviate from its initial value. The system then temporarily exits the parametric resonance region, and the accumulated energy couples into other modes, leading to a decrease in amplitude. When the amplitude drops, the natural frequency recovers, the system re‑enters the resonance region, and the amplitude grows again. This cycle repeats, resulting in a periodic amplitude modulation phenomenon. The finite element calculations indicate that the amplitude does not grow infinitely, in contrast to the theoretical prediction.

**Fig 12:**
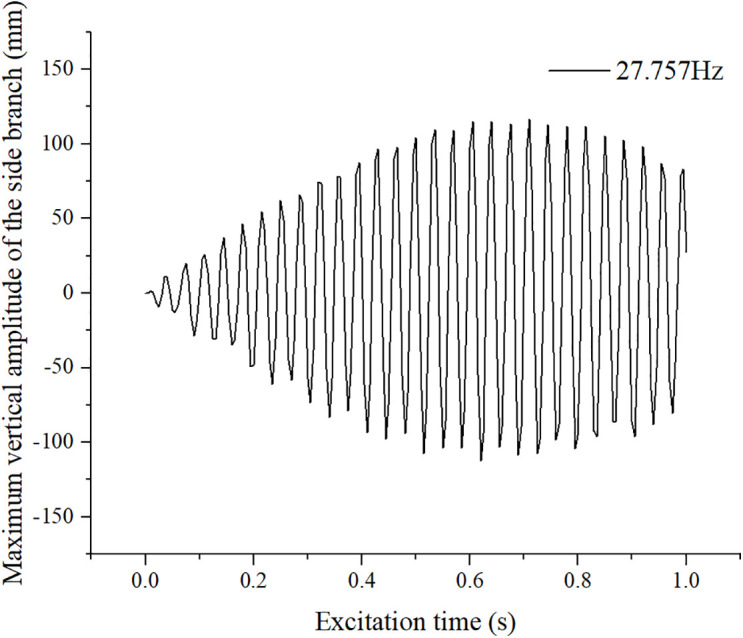
Maximum vertical amplitude of the side branch at an excitation frequency of 27.757 Hz.

To further examine the resonance behavior of the fruit branch system, an excitation with a displacement amplitude of 20 mm at the natural frequency of 27.757 Hz is applied at the base of the main stem. Under this condition, the maximum vertical amplitude of the side branch is shown in Fig 12.

As shown in Fig 12, the amplitude of the resonant response grows linearly with time, which corresponds to a typical forced oscillation response. However, when the vibration amplitude of the side branch becomes sufficiently large, the system exhibits nonlinear characteristics. When the side branch amplitude reaches a certain level, the instantaneous natural frequency of the system deviates from the external excitation frequency, causing the system to temporarily leave the resonance region, and the amplitude subsequently begins to decrease.

**Fig 13:**
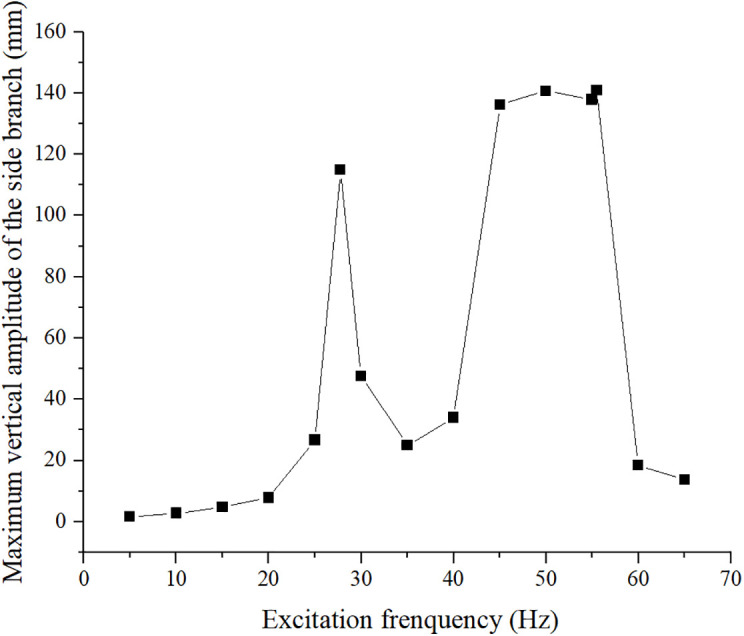
Maximum vertical amplitude of side branch under different excitation frequencies.

The dynamic responses of the fruit branch system under an excitation displacement amplitude of 3 mm and various excitation frequencies are calculated separately. The maximum vibration amplitude of the side branch in the vertical direction at each given excitation frequency is shown in Fig 13.

As shown in Fig 13, when the excitation frequency equals the natural frequency of the fruit branch system, the response amplitude of the system increases significantly. When the excitation frequency is near the parametric resonance frequency, the amplitude of the fruit branch system begins to exhibit an exponentially growing behavior, as illustrated in the frequency range of 45–55 Hz in Fig 13. Furthermore, under parametric resonance conditions, once the amplitude of the fruit branch grows to a certain threshold, the maximum amplitude of the side branch tends to stabilize and no longer increases continuously.

ABAQUS simulations further confirm that even with a small excitation amplitude, parametric resonance can still induce a large amplitude response in the system. This phenomenon directly reflects the theoretically predicted instability and has been reproduced in the simulations. Consistent with theoretical expectations, this instability is excited precisely when the excitation frequency is exactly twice the first natural frequency of the system. ABAQUS simulations demonstrate that the parametric resonance equation can accurately describe the dynamic behavior of the system in its low-order modes. The instability region predicted by the theoretical equation corresponds to the excitation conditions under which the fruit branch system exhibits a substantially large amplitude in the simulations.

### 3.3 Experimental results

The vibration amplitude of the fruit branches was influenced by several factors. inadequate clamping of the main stem leading to branch displacement, excessive curvature, abrupt changes in branch diameter, and rotation caused by branch asymmetry. These factors significantly affected the experimental results and were therefore excluded. Branches with dimensions close to the theoretically calculated values were selected. The main stem was approximately 250 mm long, and a 50 mm segment was clamped in the experiment. The diameter of the main stem was approximately 6 mm. The position of the side branch was approximately 200 mm from the base of the main stem. The side branch was 156 mm long and 2.15 mm in diameter. The excitation frequency was varied by adjusting the motor speed, and the excitation amplitude was taken as the reciprocating distance of the vibration device. The geometric parameters of the fruit branches used in the experiments are listed in Table 2.

**Table 2:** Geometric parameters of the fruit branches.

Fruit branch number	Main stem and upper stem length after clamping (mm)	Main stem diameter (mm)	Side branch length (mm)	Side branch diameter (mm)	Side branch location (mm)	Side branch angle with horizontal (°)
01	200.0	6.00	156.0	2.15	50.0	19
02	197.6	6.07	152.3	2.23	53.2	17
03	202.1	5.94	160.8	2.09	46.9	21
04	199.4	6.03	158.2	2.13	48.6	18
05	203.3	5.89	163.9	2.01	44.3	16
06	196.7	6.14	149.6	2.28	55.8	22
07	201.8	5.96	162.7	2.05	47.4	16
08	198.5	6.06	154.2	2.19	50.9	17
09	203.9	5.81	166.8	1.96	41.7	23
10	195.3	6.17	148.4	2.30	58.3	15

**Fig 14:**
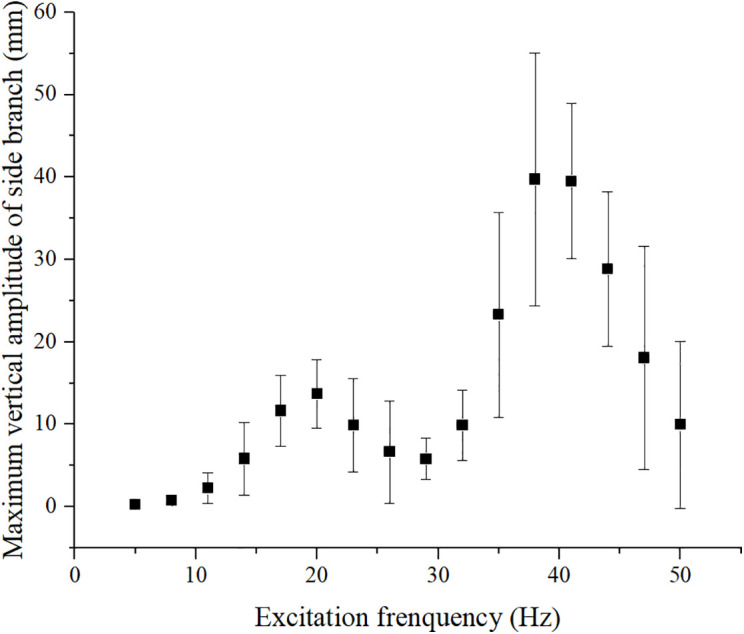
Maximum vertical amplitude of side branches at different excitation frequencies.

Vibration experiments were conducted on the test platform, and a high-speed camera recorded the vibration amplitude at the tip of the side branch. For each frequency, the maximum vertical amplitude of the side branch tip was recorded, and the standard deviation was calculated (Fig 14).

As shown in Fig 14, the amplitude-frequency characteristic curves from the experiments exhibit two distinct resonance peaks: one at a lower frequency and another at a higher frequency. This trend is consistent with that observed in Fig 13. When the excitation frequency approaches 20 Hz, most fruit branches first exhibit a resonance peak. Subsequently, as the excitation frequency approaches 40 Hz, the amplitude at the tips of the side branches reaches over 40 mm. This behavior is characteristic of principal parametric resonance.

**Fig 15:**
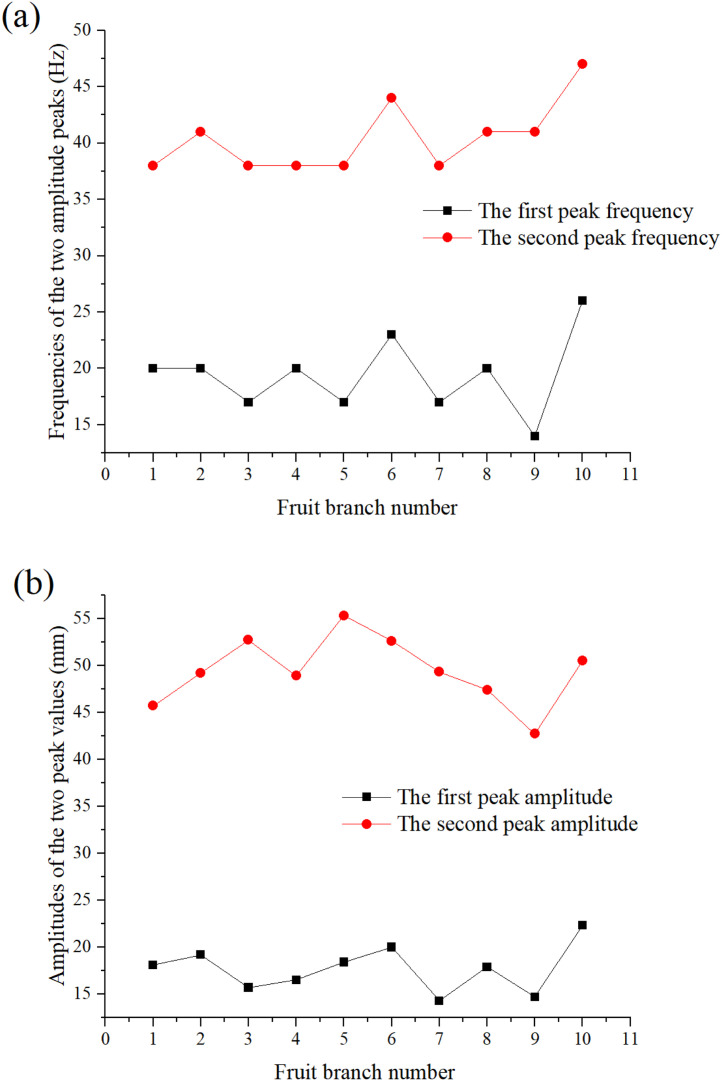
Frequency and amplitude at the vibration amplitude peak. (a) Frequency at the peak (b) Amplitude at the peak.

The frequencies and corresponding amplitudes of the two resonance peaks for each sample were extracted and plotted separately, as presented in Fig 15.

As shown in Fig 15, all samples exhibit typical parametric resonance characteristics. When the excitation frequency equals approximately the first-order natural frequency of the system, ordinary resonance occurs first. When the excitation frequency equals approximately twice the first-order natural frequency, parametric resonance occurs. Moreover, the mean value of the amplitude of the parametric resonance (49.93 ± 12.00 mm) is significantly larger than that of ordinary resonance (17.71 ± 5.54 mm). Compared with theoretical calculations and finite element simulation results, the experimentally obtained frequencies for ordinary resonance and parametric resonance are systematically lower. This deviation arises because the theoretical model assumes uniform material properties, whereas the actual side branch and main stem have different properties.

**Fig 16:**
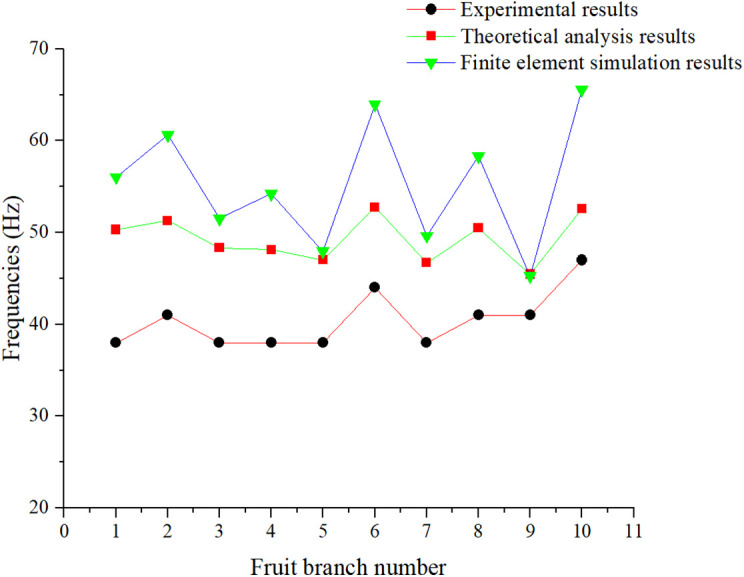
Frequency of parametric resonance of Y-shape branch by theory, finite element simulation and experiments.

To validate the theoretical and finite element results against experiments, the analytical solutions, finite element predictions, and experimentally measurements of the parametric resonance frequencies of ten fruit branches were compared. The results are shown in Fig 16.

As shown in Fig 16, the theoretical analysis agrees well with the finite element results. The theoretical model adopted here assumes zero concentrated mass at the tip, which represents the worst‑case error scenario. The frequency variation trends from the three approaches are essentially consistent, indicating that the simplified theoretical model can effectively predict the frequency characteristics of parametric resonance, thereby providing a reference for engineering practice. The experimentally measured parametric resonance frequencies are systematically lower, but their trend qualitatively matches the theoretical predictions. Possible reasons for this deviation include: the actual elastic modulus of the side branches was lower than that of the main stem, while both the theoretical and finite element models assumed identical moduli; additionally, the bark thickness was not excluded when measuring the branch diameters.

The side branch length of field‑grown blue honeysuckle trees is generally longer than 312 mm, which is more than twice the value adopted in the theoretical analysis, finite element simulation, and experiments. Since the length of the fruit branch is the primary factor affecting the natural frequency of the branch system, the natural frequency decreases significantly with increasing length. According to the trends in Fig 4 or Fig 8, when the side branch length ratio is 2, i.e., $b_{\text{3}}$ or k is taken as 2, the excitation frequency for field operation should be approximately 10–12 Hz.

As the side branch length ratio increases, the unstable region clearly shifts toward lower frequencies (see Fig 4). If the side branch is too long, its bending natural frequency will be lower than the rotational natural frequency of a branch of the same material but different length. The length ratio at which the bending frequency of one side branch equals the rotational frequency of another side branch is calculated as follows. Let the lengths of the two fruit branches be $L_{\text{1}}$ (the branch producing the second‑order frequency) and $L_{\text{2}}$ (the branch producing the first‑order frequency). According to the formula for the n ‑th natural angular frequency of a cantilever beam:


$$\begin{array}{c} \omega_{n}={\beta_{n}}^{\text{2}}\sqrt{\frac{EI_{b}}{\rho Al^{\text{4}}}} \end{array}$$ (41)

where $\beta_{n}$ is a dimensionless eigenvalue constant. From the Eq. (9), one obtains for the first order $\beta_{\text{1}}\approx 1.875$, and for the second order $\beta_{\text{2}}\approx 4.694$. Setting the second‑order bending frequency of $L_{\text{1}}$ equal to the first‑order rotational frequency of $L_{\text{2}}$, $\omega_{\text{2}}{|}_{L_{\text{1}}}=\omega_{\text{1}}{|}_{L_{\text{2}}}$, considering that the material and cross‑sectional area are the same, we obtain:


$$\begin{array}{c} \frac{L_{\text{1}}}{L_{\text{2}}}\approx 2.503 \end{array}$$ (42)

Therefore, the length ratio of the two branches is approximately 2.5. Based on the analysis, the following field management recommendations can be made.

When the clamping position is approximately 150 mm from the side branch, a side branch length of 312 mm to 2.5 times this value (i.e., up to about 780 mm) causes the fruit branch system to enter parametric resonance at an excitation frequency of approximately 10–12 Hz. For side branch lengths below 312 mm, a higher excitation frequency is required; e.g., for a 156 mm side branch, the frequency that induces parametric resonance can reach 40 Hz.

The upper stem length ratio $b_{\text{2}}$ has little influence on the position of the unstable region; however, once the upper stem length exceeds 5 times the main stem length, the unstable region narrows significantly or even disappears (see Fig 9). If the main stem length is 150 mm, the upper stem should be pruned when it exceeds 750 mm to prevent it from suppressing the parametric resonance instability.

## 4. Discussion

When the side branch is parallel to the ground and perpendicular to the excitation direction, its dynamic equation takes the same form as that of the upper stem presented in this study. In this case, the side branch vibration is forced vibration, and the dynamic equations become forced vibration equations.

For a slender and light side branch, the lowest-order mode of the system is dominated by the side branch vibration, while the amplitudes of the main stem and upper stem are relatively small. This indicates that, for a fruit branch system, the longest horizontal branch typically governs the lowest-order mode.

In the experiments, the parametric resonance amplitude was smaller than that obtained from finite element simulations. This discrepancy arises because the fruit branch material exhibits significant nonlinear damping under high-frequency excitation. Owing to this high damping, once the amplitude of parametric resonance increases, the instability region disappears and the amplitude no longer grows.

The samples used in the experiments were branches with leaves and fruits removed, which differ from actual harvesting conditions. During harvesting, factors such as leaf damping, fruit weight, and the mass reduction after fruit detachment significantly alter the instability region of the fruit branch. Therefore, harvesting machinery employing variable-frequency excitation may offer better applicability.

## 5. Conclusions

Based on the rotational mode of the fruit branch, the dynamic equations of a Y-shaped fruit branch system under external excitation were derived and simplified according to the branches angles. The simplified equations show that parametric resonance can be excited even when the excitation amplitude is small. Furthermore, the low-order natural frequencies obtained from subsequent finite element simulations and experiments agree well with the theoretical results, and the parametric resonance was accurately reproduced.

With specified branch parameters and an excitation amplitude below 30 mm, the side branch length significant influences the instability region. When the side branch length exceeds twice its initial value, the excitation frequency of the instability region decreases from 50.32 Hz to below 13 Hz. When the upper stem length exceeds 5.3 times its initial value (the initial upper stem length being approximately 1.77 times the main stem length), the excitation frequency at instability exhibits little variation, but the instability region becomes very narrow and eventually disappears. This demonstrates that branch length most strongly affects the system instability frequency, suggesting that in blue honeysuckle cultivation, controlling side branch length enables active regulation of harvest vibration characteristics.

ABAQUS simulations and experimental results show that when the fruit branch undergoes parametric resonance, the branch stiffness changes with increasing amplitude, causing the actual natural frequency to deviate from the initial natural frequency, Moreover, the unbounded amplitude growth predicted by the theoretical undamped model does not occur in a real system.

Experimental results indicate that at an excitation amplitude of 20 mm, the side branch amplitude in the parametric resonance region is amplified to 2.78 times that in the ordinary resonance region, and the peak interval width is significantly larger. Using parametric resonance greatly reduces the requirement for precise matching of the natural frequency of the fruit branch system. This conclusion provides a quantitative theoretical basis for blue honeysuckle vibration harvesting.

## Supporting information

S1 File Experimental data from blue honeysuckle branch vibration. (XLSX)
